# Progression of fragile X-associated tremor/ataxia syndrome revealed by subtype and stage inference

**DOI:** 10.1093/braincomms/fcaf483

**Published:** 2025-12-10

**Authors:** Douglas Ezra Morrison, Matthew Dominic Ponzini, Ellery R Santos, Hazel Maridith Barlahan Biag, Glenda Espinal, Flora Tassone, Susan M Rivera, David Hessl, Andrea Schneider, James A Bourgeois, Randi Hagerman, Kyoungmi Kim

**Affiliations:** Department of Public Health Sciences, School of Medicine, University of California, Davis, Davis, CA 95616, USA; Department of Public Health Sciences, School of Medicine, University of California, Davis, Davis, CA 95616, USA; MIND Institute, University of California, Davis, Sacramento, CA 95817, USA; MIND Institute, University of California, Davis, Sacramento, CA 95817, USA; Department of Pediatrics, University of California, Davis Medical Center, Sacramento, CA 95817, USA; MIND Institute, University of California, Davis, Sacramento, CA 95817, USA; MIND Institute, University of California, Davis, Sacramento, CA 95817, USA; Department of Biochemistry and Molecular Medicine, School of Medicine, University of California, Davis, Sacramento, CA 95817, USA; Department of Psychology, College of Behavioral and Social Sciences, University of Maryland, College Park, MD 20742, USA; MIND Institute, University of California, Davis, Sacramento, CA 95817, USA; Department of Psychiatry and Behavioral Sciences, University of California, Davis Medical Center, Sacramento, CA 95817, USA; MIND Institute, University of California, Davis, Sacramento, CA 95817, USA; Department of Psychiatry and Behavioral Sciences, University of California, Davis Medical Center, Sacramento, CA 95817, USA; MIND Institute, University of California, Davis, Sacramento, CA 95817, USA; Department of Pediatrics, University of California, Davis Medical Center, Sacramento, CA 95817, USA; Department of Public Health Sciences, School of Medicine, University of California, Davis, Davis, CA 95616, USA

**Keywords:** fragile X-associated tremor/ataxia syndrome, subtype and stage inference, disease development and progression, sex differences, latent subgroups

## Abstract

The fragile X-associated tremor/ataxia syndrome (FXTAS) is a neurodegenerative disorder caused by the premutation (55–200 CGG repeats) in the fragile X messenger ribonucleoprotein-1 (*FMR1*) gene. An open question is: In what sequential order do FXTAS symptoms typically appear, and how does that sequence vary among patients and between males and females? We applied the ordinal-outcomes version of the Subtype and Stage Inference algorithm (‘Ordinal SuStaIn’) to identify the sequential events of clinical and brain MRI changes in cross-sectional data collected during baseline visits from a longitudinal cohort of FXTAS patients at Stages 0–5. We included 28 neurodegenerative symptoms collected from 253 premutation carriers (101 females and 152 males) and 44 controls (7 females and 37 males), aged 40–86 years old at entry, who participated in two longitudinal studies, with entry dates between 2008 and 2023. We found substantial differences in order of events depending on sex, and possibly in combination of sex and CGG repeats. The main finding is the predominance of the psychiatric co-morbidities that occur early in females (often before the onset of tremor and ataxia) compared to males. These findings suggest that the sequence of neuropsychiatric symptoms for FXTAS is different in females compared to males, particularly for early symptoms in disease development and progression. This could lead to sex-specific modifications of the FXTAS diagnostic stages.

## Teaser

Fragile X-associated tremor/ataxia syndrome (FXTAS) is an adult-onset neurodegenerative disorder affecting fragile X gene premutation carriers. Symptoms include tremors, balance issues and dementia. Morrison *et al*. found sex-based differences in symptom order, highlighting the need for sex-specific diagnostic criteria.

## Graphical Abstract

**Graphical Abstract:**
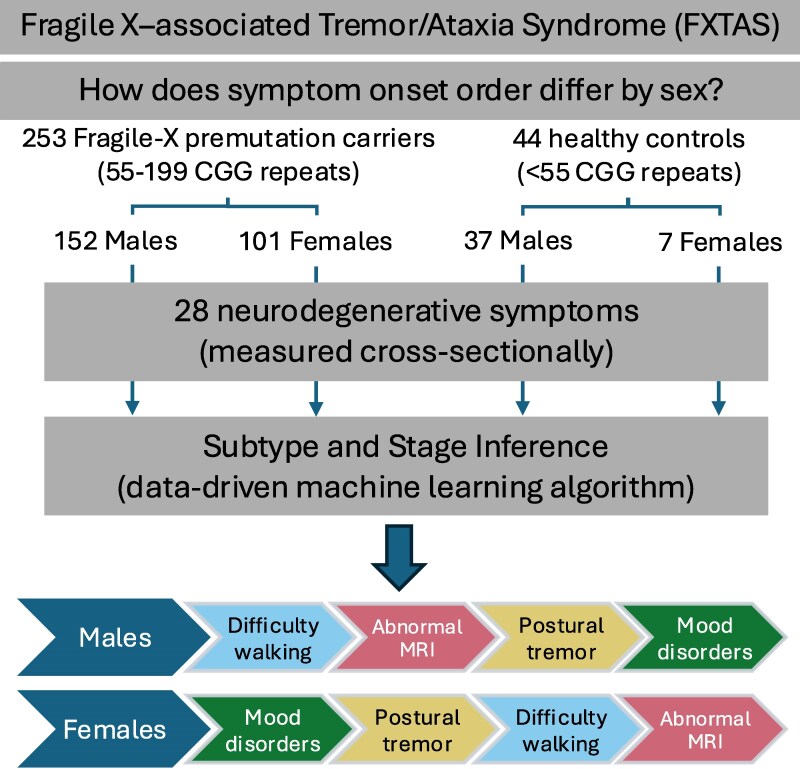

## Introduction

The fragile X-associated tremor/ataxia syndrome (FXTAS) is a neurodegenerative disorder caused by the premutation (55–200 CGG repeats) in the fragile X messenger ribonucleoprotein-1 (*FMR1*) gene.^1,2^ The premutation is associated with elevation of the *FMR1* mRNA. The RNA toxicity due to the elevated expression levels of the *FMR1* mRNA leads to oxidative stress, mitochondrial dysfunction, calcium dysregulation and sequestration of proteins important to neuronal function.^3-13^ Eosinophilic, tau-negative intranuclear inclusions are present in neurons and astrocytes throughout the CNS and in the peripheral nervous system (PNS).^14,15^ FXTAS is a late-onset disorder, usually occurring in the 60s, though some patients have an earlier onset, and its signs and symptoms worsen with age. As premutation carriers age, the prevalence of FXTAS increases from ∼40% in males in their 60s to 75% in their 80s.^16^ Females with the premutation are also at risk for FXTAS, but the prevalence is lower; ∼20% may develop FXTAS, and it is usually less severe than in males.^17^ In general, for males with FXTAS, the higher the CGG repeat number within the premutation range, the earlier onset of FXTAS and earlier death.^14^ The prevalence of the premutation is estimated at one in 148–200 females and one in 290–855 males in the general population.^18,19^

The symptoms of FXTAS appear and develop over many years in adulthood, usually after age 50, and include tremor, ataxia, neuropathy, cognitive decline and depressive and anxiety disorders.^1,2^ Both the type and severity of FXTAS symptoms vary among patients. Some patients with FXTAS have multiple symptoms that progress rapidly in severity, while others have few symptoms that remain mild over many years. The sequence in which those symptoms typically appear is still understudied, but it could help us to understand the disease process and its variability across patients. Therefore, the present study attempts to address the following questions: (i) In what sequential order do FXTAS symptoms and brain changes typically appear? (ii) How does that sequence vary between males and females? and (iii) Do specific baseline characteristics predict the order of presentation?

Here, we applied the ordinal-outcomes version of the Subtype and Stage Inference algorithm (‘Ordinal SuStaIn’)^20,21^ to model sequential orders of clinically manifest FXTAS symptoms in a cohort of FXTAS patients. SuStaIn is a data-driven analytic approach, which was originally developed to analyse cross-sectional data to simultaneously (i) estimate the ordering of disease symptomatic events and (ii) cluster study participants into latent subtypes with different event orderings. The SuStaIn modelling approach has been applied to Alzheimer’s disease,^20-23^ amyotrophic lateral sclerosis,^24^ Parkinson’s disease,^25,26^ primary tauopathies,^27^ frontotemporal dementia,^20^ multiple sclerosis,^28^ chronic obstructive pulmonary disease (COPD),^29^ and knee osteoarthritis,^30^ but not previously for FXTAS. In this study, we applied it to a cross-sectional data set of premutation carriers diagnosed with FXTAS at different clinical stages to study the sequential ordering of FXTAS symptomatic events and sex differences in sequential orders.

## Materials and methods

### Data

#### Study cohorts

Research participants were recruited between 2008 and 2023 for two longitudinal cohorts: the Genotype-Phenotype Study in Fragile X Families (‘GP’; funded by NICHD R01HD036071; PI Hagerman) and the Trajectories and Markers of Neurodegeneration in Fragile X Premutation Carriers study (‘TRAX’; funded by NINDS NS110100; PIs Hessl and Rivera) conducted at the University of California, Davis MIND Institute. A written informed consent form was obtained from all participants according to the procedures approved by the University of California, Davis Institutional Review Board.

The GP longitudinal study is an ongoing effort focused on adult male and female carriers of the *FMR1* premutation who present with neurological problems that do not meet diagnostic criteria of FXTAS or have been diagnosed with FXTAS, and healthy control individuals without the fragile X premutation, aged 40–85 at entry, with follow-up visits approximately every 2 years. For those carriers diagnosed with FXTAS at the entry, their historical symptoms were retrospectively collected and recorded.

The TRAX longitudinal study is an ongoing effort studying adult male premutation carriers ranging from 40 to 82 years and male healthy controls ages 40–75 at baseline visit. The TRAX participants return for follow-up visits with varying intervals between visits averaging 2.5 years to assess phenotypic progression over time.

In both studies, each evaluation includes a detailed medical history, neurological examination, neuropsychological testing including the Wechsler Adult Intelligence Scale, Fourth Edition (WAIS IV), Behavior Dyscontrol Scale (BDS-2), Mini-Mental State Exam (MMSE) and Cambridge Automated Neuropsychological Test Battery (CANTAB); Structured Clinical Interview for DSM-IV Disorders (SCID-I/NP), motor testing and brain MRI.^31-33^ After the clinical and MRI evaluation, each patient received a FXTAS stage designation ranging from 0 to 6 according to stages identified by Bacalman *et al*.^34^ based on tremor and ataxia severity. Stage 1 represents subclinical or uncertain tremor; Stage 2 is mild tremor without significant interference with activities of daily living (ADLs); Stage 3 is significant tremor that interferes with ADLs and significant ataxia; Stage 4 is significant ataxia needing a cane or walker; Stage 5 is requiring a wheelchair; and Stage 6 is bedridden. No participants at Stage 6 were included in the study (Table S1).

Parkinson’s disease was defined as having a diagnosis of Parkinson’s disease prior to the study enrolment. The diagnosis of Parkinson’s disease was made by the patient’s primary care physician or neurologist, outside of the study; we documented if this diagnosis was made in the medical history section of the study records.

Parkinsonian features were characterized by the presence of at least one of the following signs at examination: masked facies, increased tone, pill-rolling tremor, or stiff gait. Stiff gait means the individual lacked the normal flow of movement and looked stiff but was not spastic.

Tremor severity was assessed using elements of the Unified Parkinson’s Disease Rating Scale (UPDRS) with scores interpreted as: 0 = no tremor, 1 = minimal tremor, 2 = mild tremor noticeable during activities, 3 = moderate tremor affecting multiple tasks and 4 = severe tremor significantly impairing daily function.

Similarly, ataxia severity was evaluated using the International Cooperative Ataxia Rating Scale (ICARS) scoring, interpreted as: 0 = no ataxia, 1 = slight abnormality, 2 = moderate abnormality (noticeable but does not interfere significantly), 3 = marked impairment (function slightly affected) and 4 = severe impairment (complete inability to perform the task).^35^

The primary sequence analysis of symptomatic events included baseline visit data from 297 participants from the GP and TRAX cohorts, consisting of 253 premutation carriers and 44 controls. Table 1 provides demographic information about the study participants. Data from unaffected control participants were used to estimate the distribution of the analysed symptoms among non-FXTAS individuals as a reference in SuStaIn modelling (more details in section Ordinal SuStaIn model). Then the cases’ data were used to estimate FXTAS event sequences, using the estimated control distributions to account for random variation in observed symptoms unrelated to the underlying event sequence.

**Table 1:** Descriptive statistics of patient characteristics by sex and CGG repeat level

Characteristic	Male		Female		M versus F (all CGG combined)
	CGG <55 *n* = 37^a^	CGG ≥ 55 *n* = 152^a^	CGG <55 *n* = 7^a^	CGG ≥ 55 *n* = 101^a^	*P*-value^b^
Age at visit					
Mean (SD)	55.4 (9.87)	63.5 (9.49)	55.1 (11.49)	60.7 (11.45)	0.210^c^
Median [min, max]	54 [40, 75]	64 [40, 86]	54 [44, 78]	61 [40, 85]	
Primary race/ethnicity					
White	29 (81%)	122 (90%)	3 (60%)	76 (96%)	0.250^d^
Hispanic	4 (11%)	7 (5.1%)	0 (0%)	1 (1.3%)	
Black	2 (5.6%)	0 (0%)	1 (20%)	0 (0%)	
Other	1 (2.8%)	7 (5.1%)	1 (20%)	2 (2.5%)	
Missing	1 (2.7%)	16 (11%)	2 (29%)	22 (22%)	
FXTAS stage					
0	36 (100%)	30 (20%)	3 (100%)	30 (37%)	0.454^d^
1	0 (0%)	28 (19%)	0 (0%)	6 (7.4%)	
2	0 (0%)	28 (19%)	0 (0%)	15 (19%)	
3	0 (0%)	39 (27%)	0 (0%)	22 (27%)	
4	0 (0%)	16 (11%)	0 (0%)	5 (6.2%)	
5	0 (0%)	6 (4.1%)	0 (0%)	3 (3.7%)	
Missing	1 (2.7%)	5 (3.3%)	4 (57%)	20 (20%)	
CGG repeats					
Mean (SD)	29.3 (6.07)	86.9 (19.80)	33.6 (8.18)	87.0 (19.60)	0.057^c^
Median [min, max]	30 [20, 47]	85 [55, 185]	31 [29, 52]	86 [55, 167]	

^a^ *n* (%). ^b^*P*-values represent tests for sex differences in distributions of characteristics, all CGG repeat levels. ^c^*P*-value for significance of sex difference by Wilcoxon rank sum test. ^d^*P*-value for significance of sex difference by Fisher's exact test.

#### Symptoms of neurodegenerative events

We analysed measurements of 28 ordinal symptoms, each with between two and six levels, listed in Table 2. Herein, the term ‘symptoms’ refers to a broad range of medical signs or indications of medical state observed from patients. Each ‘clinically elevated’ ordinal level (above the first-listed, reference level) constitutes an outcome event in the disease progression modelling analysis (see section Statistical analysis). Abbreviations used in symptom names are listed in Table 3.

**Table 2:** Symptoms included in analysis

Category	Symptom	Defined ordered levels	Female (%)^a^	Male (%)^a^	*P*-value^b^
Ataxia	Tandem walk	Normal, abnormal (<10 steps), unable (absent)	50.7	79.7	<0.001
Ataxia	Ataxia severity	0, 1, 2, 3, 4	32.6	31.4	0.888
CANTAB	paired associates learning (PAL): total errors	≤13, > 13	71.4	65.9	0.803
CANTAB	Reaction time (RTI): five-choice movement time	≤368.57, > 368.57	27.3	32.3	0.805
CANTAB	Spatial Working Memory (SWM): between errors	≤26, > 26	10	38.2	0.020
MRI	Corpus callosum thickness	Normal, Thin	23.1	45.3	0.059
MRI	Genu white matter hyperintensity	No, Yes	41.7	50	0.629
MRI	Middle cerebellar peduncle (MCP): white matter hyperintensity	None, Mild, Moderate/Severe	0	39.3	<0.001
MRI	MRI: cerebellar	None, mild, moderate/severe	50	65.1	0.176
MRI	MRI: cerebral	None, mild, moderate/severe	92.6	81.6	0.234
MRI	Splenium white matter hyperintensity	None, mild, moderate/severe	66.7	66.7	>0.999
Parkinson's disease	Parkinson's disease	No, Yes	21.1	16.7	0.720
Parkinson's disease	Parkinsonian features	No, yes	15.1	23	0.209
SCID	Anxiety disorders	Absent/sub-threshold, threshold	76.4	44.1	<0.001
SCID	Mood disorders	Absent/sub-threshold, threshold	63.9	32.2	<0.001
SCID	Somatoform disorders	Absent/sub-threshold, threshold	11.3	1.4	0.003
SCID	Substance use disorders	Absent/sub-threshold, threshold	12.5	24.6	0.048
Scores	Behavior Dyscontrol Scale—2nd Edition (BDS-2): total score	≥20, < 20	22.1	31.9	0.129
Scores	Mini-Mental State Examination (MMSE): total score	Normal (26–30), mild impairment (20–25), moderate impairment (10–19)	5.3	20	0.010
Thyroid	Hyperthyroid	No, yes	2.9	1.7	0.632
Thyroid	Hypothyroid	No, yes	20.8	11.7	0.104
Thyroid	Autoimmune diagnoses or symptoms	No, yes	20.7	7.7	0.004
Tremors	Head tremor	No, yes	45.2	22.2	0.018
Tremors	Intention tremor	No, yes	60.2	53.9	0.348
Tremors	Intermittent tremor	No, yes	28.6	26.4	0.741
Tremors	Postural tremor	No, yes	47.5	37.3	0.164
Tremors	Resting tremor	No, yes	20.7	18	0.609

The first level in each cell of the ‘Defined ordered levels’ column is the ‘reference level’ for the corresponding symptom, and subsequent levels are considered ‘clinically elevated levels’. Columns ‘Female’ and ‘Male’ list percentages of clinically elevated levels at baseline visit, stratified by sex.

^a^% of participants with clinically elevated levels. ^b^*P*-value for significance of sex difference by Fisher's exact test.

**Table 3:** Abbreviations used in article

Abbreviation	Meaning
ADLs	Activities of daily living
BDS-2	Behavior Dyscontrol Scale—Second Edition
CANTAB	Cambridge Neuropsychological Test Automated Battery
CC	Corpus callosum
DSM	Diagnostic and Statistical Manual of Mental Disorders
EF	Executive function
FXTAS	Fragile X-associated tremor/ataxia syndrome
GP	Genotype–phenotype study in fragile X families
Hyp.	Hyperintensity
ICARS	International Cooperative Ataxia Rating Scale
MCP	Middle cerebellar peduncle
MMSE	Mini-Mental State Exam
Mod.	Moderate
MRI	Magnetic resonance imaging
OS/OSA	Ordinal SuStaIn algorithm
PAL	paired associates learning
PD	Parkinson's disease
RTI	Reaction time
SCID	Structured Clinical Interview for DSM Disorders
SuStaIn	Subtype and Stage Inference
SWM	Spatial working memory
TRAX	Trajectories and Markers of Neurodegeneration in Fragile X Premutation Carriers study
UPDRS	Unified Parkinson’s Disease Rating Scale
WM	White matter
WAIS IV	Wechsler Adult Intelligence Scale (Fourth Edition)

We created a composite variable named ‘autoimmune diagnoses or symptoms’ combining systemic lupus erythematosus, rheumatoid arthritis, multiple sclerosis, positive anti-nuclear antibody (ANA), Sjogren’s syndrome and Raynaud’s syndrome, since these conditions were too rare to analyse separately (details in Supplementary material, autoimmune diagnoses or symptoms). We also created composite variables for four domains of the Structured Clinical Interview for DSM-IV (SCID-IV): mood disorders, substance use disorders, anxiety disorders and somatoform disorders; there were no participants with psychotic disorders in our data (more details in Supplementary material, SCID composite variables). We also created composite variables for MRI variables for cerebral and cerebellar abnormalities; we did not combine the variables representing corpus callosum MRI abnormalities, since these variables were created using Likert scales that differed from each other (details in Supplementary material, MRI variables). For the Cambridge Neuropsychological Test Automated Battery (CANTAB) variables, we used categorization cutoffs taken from Talebi *et al*.^36^

The developers of the Ordinal SuStaIn algorithm recommend a minimum of three observations per symptom level.^21^ In order to limit the number of symptom levels with extremely rare observations in our models, we combined the ‘Moderate’ and ‘Severe’ levels for the MRI variables and the ‘Absent’ and ‘Sub-Threshold’ levels for the SCID domain variables.

Tables S1–S13 summarize each of the symptoms that we included in the analysis models, stratified by CGG repeat size (CGG <55, CGG 55–99, CGG 100–199) and sex.

### Statistical analysis

#### Ordinal SuStaIn model

We applied a discrete event-based disease progression model^37-39^ to our data using the Ordinal Subtype and Stage Inference (‘SuStaIn’) algorithm^21^ to estimate event orderings and subtypes for FXTAS patients. SuStaIn is a data-driven statistical modelling algorithm that combines event-based disease progression modelling^37-39^ and latent-cluster finite mixture modeling^40-42^ to model event sequences using cross-sectional samples of patient and control populations. The algorithm simultaneously clusters individuals into latent subtypes and characterizes the event ordering that best defines each subtype, thus capturing heterogeneity in both disease subtype and disease stage.

Ordinal SuStaIn^21^ is a version of the SuStaIn modelling algorithm,^20^ adapted for analysing ordinal-valued data. Ordinal SuStaIn uses a ‘scored events model’, which assumes that for each symptom, there is a discrete set of underlying ordinal severity levels, but the measured versions of the symptoms may contain some amount of random noise. For example, a patient who was really at ataxia severity level 2 may be incorrectly assessed as being at ataxia severity level 1, depending on the patient’s temporary disease status on the day of the exam or inter-rater differences. The first step in applying the Ordinal SuStaIn algorithm is to determine, for each symptom, the probability that an individual is ‘correctly scored’ at their ‘true underlying level.’^21^ Following the procedures proposed in the original SuStaIn methodology papers,^20,21^ we used the distributions of symptoms observed among the controls to estimate the rates at which premutation carriers experience symptoms, and we thus estimated the percentages of FXTAS-related symptoms (i.e. the proportion of symptoms due to FXTAS among premutation carriers) as the percentage of controls who were assessed as being at the reference level (Table S14). We limited the estimated probability of correct scoring to 95% in order to allow for a plausible probability of symptoms unrelated to FXTAS progression.

Ordinal SuStaIn then uses Markov Chain Monte Carlo (MCMC) sampling^43^ to estimate the Bayesian posterior probability of each possible event sequence for each subtype given the training dataset, assuming a uniform prior distribution over the set of all possible orderings. We conducted subgroup analyses by fitting models stratified by sex (section ‘Sex differences in sequential orders’), CGG repeats (<100 versus ≥100) (Supplementary material, analyses stratified by CGG repeats) and combinations of sex and CGG repeats (<100 versus ≥100) (Supplementary material, Comparing sexes stratified by CGG level). In these subgroup analyses, we did not search for latent clusters.

#### Imputation of missing data

We assumed that missing symptom data were missing at random (MAR).^44,45^ As the longitudinal cohorts evolved over time, new instruments were adopted and added; much of the missingness was due to adding additional measures to the study protocols in later time. The MAR assumption thus seems plausible. We substituted missing outcome event data by assigning a probability distribution across the possible values of the missing variable that matched the marginal distribution of observed data among the cases. For example, 29 of the 253 cases (11.5%) had missing values for ataxia severity, and 224 (88.5%) had recorded values, distributed among ataxia severity levels 0–4 (143 (63.8%), 37 (16.5%), 19 (8.5%), 16 (7.1%) and 9 (4%), respectively). For the 29 cases with missing values, we assigned probabilities of 63.8%, 16.5%, 8.5%, 7.1% and 4.0% to ataxia severity levels 0, 1, 2, 3 and 4, respectively, and missing values were imputed under the marginal distribution of the observed data.

#### Statistical hypothesis tests

To test for statistical significant evidence of differences in event sequences between males and females and between lower (CGG <100) and higher CGG repeats (CGG 100–199), we implemented a permutation test^46^ to calculate a *P*-value that is the probability of observing a difference at least as extreme as the test statistic given that the null hypothesis of no difference is true. We first created 1000 permuted datasets in which we randomly shuffled the variable being tested. We computed the mean log-likelihood of the data for each permuted dataset (averaging across MCMC samples and summing across the strata being compared) and compared the distribution of permuted mean log-likelihoods to the observed log-likelihood calculated from the original (unpermuted) dataset. We computed the empirical *P*-value by first computing the percentile of the observed mean log-likelihood relative to the empirical distribution of the permuted mean log-likelihoods, subtracting that percentile from 1 if larger than 0.5 and then multiplying by two to calculate a two-sided test statistic. We declared significance if the *P*-value was ≤0.05.

#### Latent subtype clustering

We also fitted the model on the full dataset (not stratified by sex or CGG) for 2–12 latent subtypes, each with its own ordering. We determined the optimal number of latent subtypes for this dataset using the cross-validation information criterion (CVIC) and the out-of-fold log-likelihood (OOFLL) criterion^20^; both criteria quantify how well models containing a given number of subtypes extend to new data not used in training. We performed 10-fold cross-validation on the unstratified data and calculated the CVIC and OOFLL for 1–12 latent subtypes. We selected an optimal number of latent subtypes based on a combination of these metrics and the principle of parsimony (‘Occam’s razor’). Once we determined the optimal number of latent subtypes, we classified each individual into their most likely subtype and investigated distributional differences of sex and CGG repeat size between the subtypes (Table 4).

**Table 4:** Demographics of 4 latent subtype clusters identified by Ordinal SuStaIn

Characteristic	Overall *n* = 214^a^	Subtype 1 *n* = 74^b^	Subtype 2 *n* = 49^b^	Subtype 3 *n* = 32^b^	Subtype 4 *n* = 59^b^	*P*-value
CGG repeats, Mean (SD)	87.8 (18.9)	92.9 (22.1)	87.5 (18.2)	85.2 (16.3)	83.1 (15.2)	0.029^c^
Age at visit, mean (SD)	64 (10)	62 (11)	63 (10)	65 (7)	66 (9)	0.072^c^
Male, *n* (%)	137 (64%)	49 (66%)	30 (61%)	21 (66%)	37 (63%)	0.940^d^
Primary race/ethnicity, *n* (%)						
White	174 (92.1%)	59 (92.2%)	40 (93.0%)	29 (93.5%)	46 (90.2%)	0.875^e^
Hispanic	7 (3.7%)	2 (3.1%)	1 (2.3%)	2 (6.5%)	2 (3.9%)	
Other	8 (4.2%)	3 (4.7%)	2 (4.7%)	0 (0.0%)	3 (5.9%)	
Missing	25	10	6	1	8	

^a^ *n* (column %); 39 carriers had experienced too few events to be accurately classified into a subtype and were excluded from these results. ^b^*n* (column %). ^c^Group comparison was done by One-way analysis of means (not assuming equal variances). ^d^Group comparison was done by Pearson's chi-squared test. ^e^Group comparison was done by Fisher's exact test.

#### Visualizing modelling results

We visualized the results of Ordinal SuStaIn analysis using ‘positional variance diagrams’ (PVDs).^37,38^ PVDs are heatmaps with symptomatic events on the *y*-axis and sequence positions of events on the *x*-axis. Each event is estimated to occur at some point in continuing time *relative to* other events in sequence. It should be noted that the exact onset time of each event cannot be determined by the method we used for this analysis. The PVD’s colour scale indicates the Bayesian posterior probability that a particular event (*y*-axis) appears at a particular position along the progression sequence (*x*-axis). The heatmap colours (red, blue, magenta, orange, purple) indicate the ordinal levels of symptom progression. Colour intensity represents the Bayesian posterior probability of sequence position. That is, a brighter colour indicates a more probable sequence position, and a paler colour indicates a less probable position.

We visualized differences in event sequences by sex, CGG repeat size, or latent subgroup using ‘positional difference graphs’ (PDGs). PDGs consist of two juxtaposed lists of symptoms in each of two subgroups, with each list ordered by most likely sequence position in that subgroup. Lines connect the locations of the same symptoms between the subgroups. The colours of the connecting lines indicate whether the event occurs at an earlier position in the right-hand subgroup compared to the left-hand subgroup (blue line), at a later position (red line), or at the same position (grey line). FXTAS stages are connected by black lines to highlight which symptoms occur in different FXTAS stages between the two subgroups (i.e. which symptom lines cross one or more stage lines).

### Software

The Ordinal Sustain analysis was performed using the Python programming language, version 3.9,^47^ with the pySuStaIn package.^48^ Data pre-processing and results post-processing and visualization were performed in R version 4.5.1 (13 June 2025)^49^ using the tidyverse packages.^50^ The reticulate package^51^ was used to create an application programming interface between Python and R. A colour-blind friendly colour palette was selected using the rcartocolor and colorblindcheck R packages.^52,53^ The code used to perform this analysis is available through a repository at https://github.com/UCD-IDDRC/fxtas.

## Results

Table 1 describes summary statistics of patient characteristics included in the analysis. Our data included: 253 fragile X premutation carriers and 44 controls; 189 males and 108 females; and 60, 34, 43, 61, 21 and 9 premutation carriers at FXTAS stages 0, 1, 2, 3, 4 and 5, respectively. 40% of premutation carriers were at FXTAS Stages 3–5 at the time of their baseline visit (Table 1). Out of 180 premutation carriers who were screened for parkinsonian features, 34 carriers (47.9% of 71) at FXTAS Stages 3–5 had parkinsonian features, while 8 premutation carriers (7.5% of 107) at FXTAS Stages 0–2 had parkinsonian features (Table S6). Nine premutation carriers (5 male and 4 female) were diagnosed with Parkinson’s disease by the participants’ primary care physicians or neurologists, whereas 43 premutation carriers (32 male and 11 female) had parkinsonian features assessed as part of the study protocols (Table S5).

We found no differences between males and females in age at baseline visit, ethnicity/race, FXTAS stage, or CGG repeat length. Table 2 lists the symptoms and their ordinal levels and reports percentages of clinically elevated levels at baseline visit, stratified by sex. We found significant sex differences in percentages of clinically elevated (non-reference) symptom levels at baseline visit for ‘tandem walk’ (50.7% of females versus 79.7% of males, *P*-value < 0.001), ‘spatial Working Memory (SWM): between errors’ (10% of females versus 38.2% of males, *P*-value = 0.020), ‘Middle cerebellar peduncle (MCP): white matter hyperintensity’ (0% of females versus 39.3% of males, *P*-value < 0.001), ‘anxiety disorders’ (76.4% of females versus 44.1% of males, *P*-value < 0.001), ‘mood disorders’ (63.9% of females versus 32.2% of males, *P*-value < 0.001), ‘somatoform disorders’ (11.3% of females versus 1.4% of males, *P*-value = 0.003), ‘substance use disorders’ (12.5% of females versus 24.6% of males, *P*-value = 0.048), ‘Mini-Mental State Examination (MMSE): total score’ (5.3% of females versus 20% of males, *P*-value = 0.010), ‘autoimmune diagnoses or symptoms’ (20.7% of females versus 7.7% of males, *P*-value = 0.004) and ‘head tremor’ (45.2% of females versus 22.2% of males, *P*-value = 0.018).

### Sex differences in sequential orders

**Figure 1:**
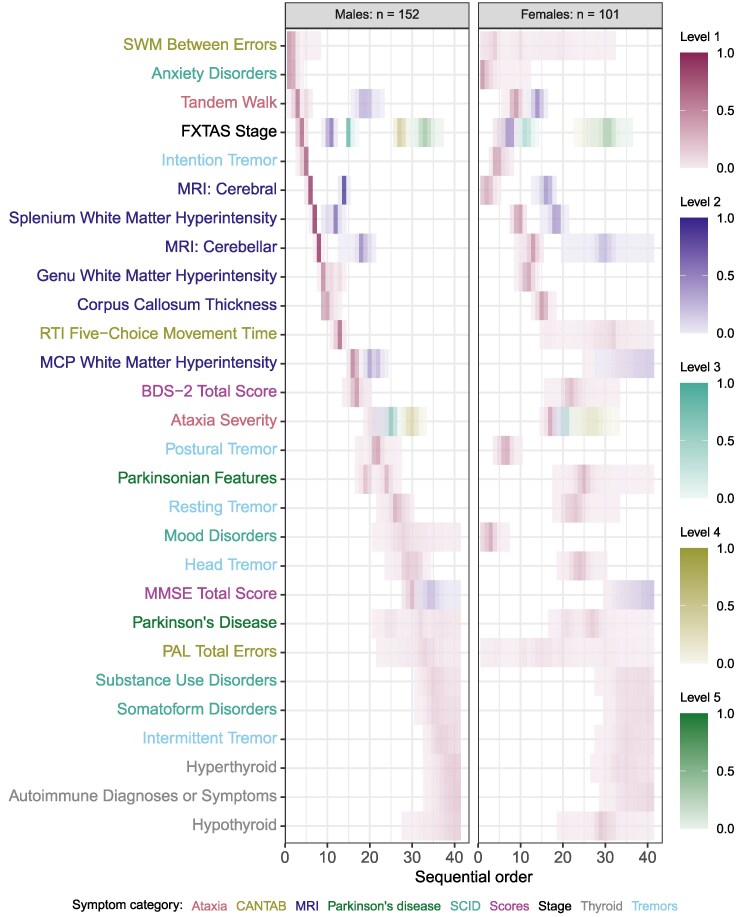
**Event sequences of FXTAS symptoms stratified by sex.** The heatmap colours (violet red, royal blue, cadet blue, khaki, forest green) indicate the ordinal levels of symptom progression (Table 2). Heatmap colour gradient intensity represents the Bayesian posterior probability of the sequence position; the brighter the colour, the more probable that the corresponding symptom event occurs in that position in the sequence. Label text colours indicate symptom categories (Table 2). Abbreviations: BDS-2, Behavior Dyscontrol Scale—Second Edition; CANTAB, Cambridge Neuropsychological Test Automated Battery; FXTAS, fragile X-associated tremor/ataxia syndrome; MCP, middle cerebellar peduncle; MMSE, Mini-Mental State Exam; Mod., moderate; MRI, magnetic resonance imaging; PAL, paired associates learning; RTI, reaction time; SCID, Structured Clinical Interview for DSM Disorders; SWM, Spatial Working Memory. Permutation test statistic (log-likelihood): −5323.03; *P*-value < 0.001 (*n* = 253 premutation carriers).

**Figure 2:**
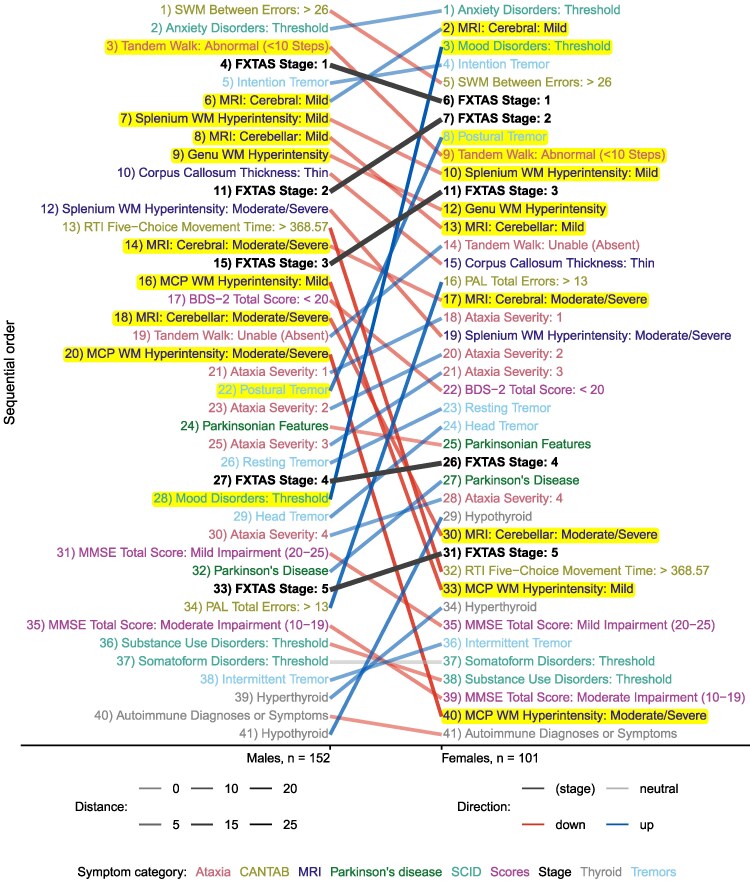
**Positional differences in estimated event sequence between males and females.** Red lines indicate symptoms that moved to later positions between the left-hand subgroup and the right-hand subgroup. Blue lines indicate symptoms that moved to earlier positions. Light grey lines indicate symptoms that did not change positions. Black lines indicate FXTAS stages. Line opacity levels indicate the number of positions changed (higher opacity represents more positions changed). Label text colours indicate symptom categories (Table 2). Yellow highlights indicate symptoms with clinically significant positional differences between subgroups. Abbreviations: BDS-2, Behavior Dyscontrol Scale—Second Edition; CANTAB, Cambridge Neuropsychological Test Automated Battery; FXTAS, fragile X-associated tremor/ataxia syndrome; Hyp., hyperintensity; MCP, middle cerebellar peduncle; MMSE, Mini-Mental State Exam; Mod., moderate; MRI, magnetic resonance imaging; PAL, paired associates learning; RTI, reaction time; SCID, Structured Clinical Interview for DSM Disorders; SWM, Spatial Working Memory; WM, white matter. Permutation test statistic (log-likelihood): −5323.03; *P*-value < 0.001 (*n* = 253 premutation carriers).

We first characterized sequential orders of FXTAS symptoms by sex and sex differences in the estimated sequential orders. Figure 1 shows the estimated sequential orders of FXTAS symptoms in males and females, and Fig. 2 highlights sex differences in those orders. Figure S1 shows the number of premutation carriers at each disease progression stage, stratified by sex. We found statistically significant evidence of a difference in sequences of symptomatic events between males and females (*P* < 0.001). It appears that female premutation carriers developed mood disorder symptoms prior to FXTAS Stage 1 diagnosis, whereas male carriers experienced these symptoms much later (after FXTAS Stage 4) (Fig. 2). Postural tremors developed between Stage 2 and 3 for females, and between Stages 3 and 4 for males. Several MRI biomarkers of decline also appear to begin at earlier FXTAS stages for males, except for mild MRI cerebral biomarkers, which began earlier in females.

To study whether observed sex differences were modulated by CGG repeats, we conducted subgroup analyses by CGG repeat size. A statistically significant difference between males and females was found among those with lower CGG repeats < 100 (*P* = 0.022; Figs S2 and S3), while a difference between males and females was not statistically significant among those with higher CGG repeats ≥ 100 (*P* = 0.178; Figs S4 and S5). Several psychiatric disorders (as assessed by SCID) occurred prior to FXTAS Stage 1 among females with < 100 CGG repeats, but at later FXTAS stages for males with < 100 CGG repeats (Fig. S3). MRI biomarkers appeared to occur at later stages in females compared to males.

We did not find significant evidence of an overall difference between CGG <100 and CGG ≥100 (*P* = 0.16) (Figs S6 and S7). We also did not find significant evidence of an overall difference between CGG <100 and CGG ≥100 among males (*P* = 0.38) (Fig. S8). SCID anxiety disorders and SWM between errors occurred prior to Stage 1 (1st and 2nd in sequence) in males with CGG repeats <100, whereas they occurred between stage 4 and 5 (32nd and 27th in sequence) in males with CGG repeats ≥ 100 (Fig. S9). Postural, resting and intermittent tremor occurred in earlier FXTAS stages in those with CGG repeats <100 compared to those with CGG repeats ≥100. SCID substance use disorders and somatoform disorders and MRI biomarkers occurred later in the event sequence in participants with CGG repeats <100 compared to those with CGG repeats ≥100. We also did not find a significant difference between CGG <100 and CGG ≥100 among females (*P* = 0.73) (Figs S10 and S11). It should be noted that this comparison lacked statistical power given the limited sample size of only 25 females with CGG ≥100.

### Subtype clustering

We conducted latent subtype classification analysis to cluster participants that are relatively homogeneous within the same cluster and heterogeneous from other clusters based on similarities and differences in sequential patterns. The cross-validation procedure (Supplementary material, Detecting latent subtypes) suggested 11 subtypes to be the optimal number for the full, unstratified dataset (Fig. S12). The OOFLL (Supplementary material, detecting latent subtypes) showed substantial fold-to-fold variation (Fig. S13). Between 4 and 12 latent subtypes, the distribution of OOFLL appeared to be approximately unchanging and the CVIC appeared approximately flat, and thus for clinical interpretation and in consideration of the principle of parsimony, we chose to classify participants into four subtypes.

**Figure 3:**
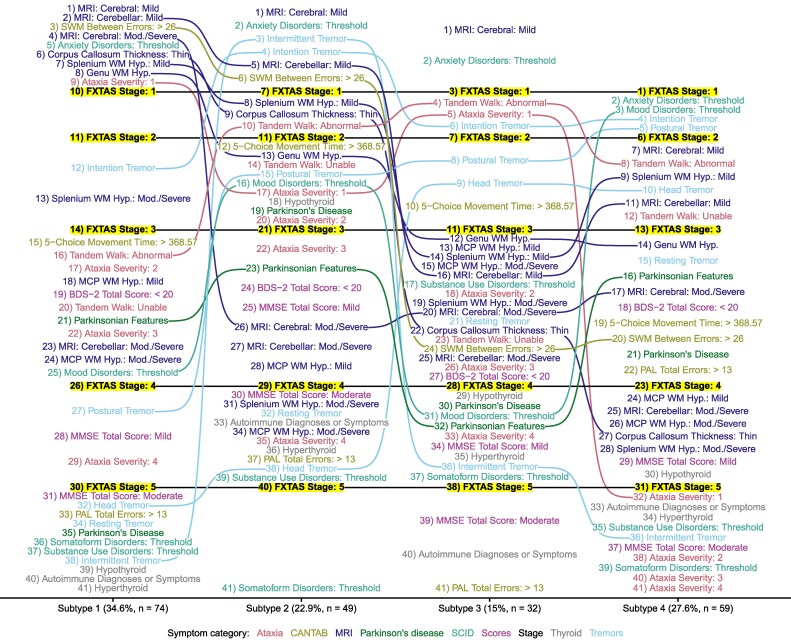
**Differences in event sequences between latent subtypes, aligned by FXTAS stage.** Lines are used to show patterns of clinical interest. Label text colours indicate symptom categories (Table 2). Abbreviations: BDS-2, Behavior Dyscontrol Scale—Second Edition; CANTAB, Cambridge Neuropsychological Test Automated Battery; FXTAS, fragile X-associated tremor/ataxia syndrome; Hyp., hyperintensity; MCP, middle cerebellar peduncle; MMSE, Mini-Mental State Exam; Mod., moderate; MRI, magnetic resonance imaging; PAL, paired associates learning; RTI, reaction time; SCID, Structured Clinical Interview for DSM Disorders; SWM, Spatial Working Memory; WM, white matter. Mean CGG repeats differed significantly between subtypes; one-way ANOVA F test statistic: 3.13 (DFs: 3 and 101.2); *P*-value = 0.029 (*n* = 214 premutation carriers; 39 carriers had experienced too few events to be accurately classified into a subtype and were excluded from these results).

Figure S14 shows the estimated sequential event orders for each of the subtype clusters. Figure 3 shows differences in event sequences between subtypes. Table 4 shows the demographics of the patients clustered in each subtype. Thirty-nine patients had experienced too few events to be accurately classified into a subtype and were excluded from Table 4. Figure S15 shows the number of premutation carriers at each disease progression stage, stratified by subtype. Subtype clustering analysis showed that CGG repeat size, a known risk factor of FXTAS, contributed to the segregation of subtypes and variations between subtypes (*P*-value = 0.029). We labelled the subtypes in descending order of mean CGG repeats. Individuals with higher CGG repeats were over-represented in Subtype 1 (CGG repeats mean = 92.9 ± 22.1) compared to the overall study population (CGG repeats mean = 87.8 ± 18.9) and other subtypes. Subtype 2 had CGG repeats close to the overall study population (87.5 ± 18.2). Subtype 3 had fewer CGG repeats (85.2 ± 16.3) than the overall study population, and Subtype 4 had the fewest CGG repeats (83.1 ± 15.2).

Using the FXTAS Stage transitions as reference points, we explored the differences in the appearing symptoms relative to these FXTAS Stage transitions. Roughly speaking: Subtype 1 appeared to experience white matter disease phenotypes earlier (before FXTAS Stage 1) compared to the other subtypes. Subtype 2 appeared to experience early intermittent tremors and intention tremors (before FXTAS Stage 1). Subtypes 1 and 2 appeared to experience memory impairment (as assessed by SWM) prior to FXTAS Stage 1, earlier than Subtypes 3 and 4, who appeared to experience memory impairment between FXTAS Stages 3–4. Subtypes 1 and 2 might be described as ‘memory-early.’ Subtype 3 appeared to experience early ataxia (between FXTAS Stages 1 and 2) and tremor onsets (between FXTAS Stages 1 and 3). This subtype had the fewest participants and may represent a mixture of smaller latent subtypes and/or include outlier individuals with unusual event sequences. Subtype 4 appeared to have a later onset of the included symptoms, with none prior to FXTAS Stage 1.

## Discussion

This study attempted to arrange the eventual symptoms of FXTAS in their possible appearing sequential orders and determine risk factors that impact on the sequential order of presentation among patients. The main findings of this analysis were substantial differences in sequential order of eventual symptoms between males and females and in combination of sex and CGG repeat size. An important general conclusion of the study is that there is substantial variation across premutation carriers in the sequence of emergence of symptoms associated with FXTAS. These findings are summarized in Table 5.

**Table 5:** Summary of main findings

Comparison groups	Outcome	Finding
Males versus females	Outcome sequence	Significant differences (*P* < 0.001, Fig. 2); psychiatric co-morbidities occur earlier in females
Latent subtypes	Outcome sequence	Significant differences in CGG repeats (*P* = 0.029, Table 3, Fig. 3); CGG repeats could be the primary determinant of heterogeneity of FXTAS phenotype and subtypes of disease

The prevalences of lifetime anxiety disorders, mood disorders and somatoform disorders in this study cohort are higher than usual population norms for these illnesses, as noted in previous studies.^54-57^ This study design did not fractionate specific lifetime psychiatric illnesses (e.g. major depressive disorder, panic disorder, somatization disorder), but these *overall* lifetime prevalence figures are notable. Prior studies of mood and anxiety disorders have demonstrated a higher-than-expected rate of these illnesses in premutation carriers.^58,59^ The same increase in lifetime psychiatric illness burden is seen in other neurodegenerative illnesses (e.g. Parkinson’s disease, multiple sclerosis).^60-68^ As many psychiatric illnesses have a strong genetic predisposition, the lifetime prevalences of psychiatric illness in this study persuasively suggest that the premutation carrier state *itself* increases the risk of psychiatric illness.

As the SCID assesses *lifetime* illness risk (as well as current illness), it is probable that the index episode of psychiatric illness *antedated* the onset of tremor, ataxia and major neurocognitive disorder seen in more advanced premutation conditions like FXTAS. As such, the high lifetime burden of psychiatric illness in this population cannot be plausibly attributed solely (perhaps substantially) to an ‘emotional reaction’ to the loss of function associated with these functional impairments but rather should be understood as at least partially a component of the premutation carrier state *itself*. It is likely that this intrinsic vulnerability to psychiatric illness is a significant component of the carrier state.

Furthermore, as only a small proportion of the patients had MMSE scores in the impaired range, it is also unlikely that the high rates of depressive and anxiety disorders (and other psychiatric illnesses) could be attributed *solely* to complications of FXTAS dementia, as most patients had normal MMSE scores. For example, depressive disorders are a common psychiatric complication of dementia, but few of the depressive disorders found here could be attributed to dementia, as they antedated any evidence of cognitive disorders. A common sequence of illnesses seen clinically in carriers is depressive and/or anxiety disorders with young to middle age adult onset, then tremor and ataxia in the 40s or 50s, followed by FXTAS dementia in the 60s or later.^59,69^ Seen in this light, and knowing the ultimate CNS vulnerability of the carrier state, retrospectively the initial presentation of depressive and/or anxiety disorders could be regarded as a psychiatric prodrome of later full-spectrum illness. This is analogous to the depressive or psychotic illness seen in SLE before rheumatologic findings and the depressive disorder that is commonly seen in the 5 years before the motor signs leading to a diagnosis of multiple sclerosis.

These data show more significant motor involvement in males than females, as predicted, but a remarkable finding is the predominance of the psychiatric illnesses that occur early in females and often before the onset of tremor and ataxia compared to males. These symptoms, particularly mood disorders, can often occur even before the diagnosis of FXTAS in females, and the stress involved with mood disorders may be a precipitating feature for the onset of FXTAS. In addition, females may experience early cognitive impairment, particularly involving executive dysfunction and memory (as measured by the BDS-2 total score and the CANTAB SWM instrument, respectively). From a clinical perspective, we have seen significant psychiatric illness comorbid with some cognitive deficits in memory and EF abilities, but these females may not meet the criteria of FXTAS diagnosis, because of the absence of significant tremor and/or ataxia. However, if white matter disease emerges on MRI, particularly in the splenium of the corpus callosum or in the periventricular area, then this demonstrates the onset of the neuropathology of FXTAS, even if there is no tremor or ataxia yet.^70-72^

Others have written about early executive function (EF) and possibly memory problems in females with the premutation who are asymptomatic for the motor features of FXTAS.^73^ In addition, Hessl *et al*.^74^ found that the Metacognition Index, which includes working memory and other EF measures, was significantly worse than controls in male carriers before the onset of FXTAS. As noted above, the psychiatric co-morbidities such as anxiety and depressive disorders can intensify before the onset of FXTAS in female carriers.

Although no neuropathological studies have examined the splenium of the corpus callosum (CCS), several *in vivo* MRI studies have reported signal changes in the CCS in patients with FXTAS. Apartis *et al*. ^70^ was the first study reporting that CCS hyperintensity occurred as frequently as middle cerebellar peduncle (MCP) hyperintensity in 22 patients with FXTAS (4 women, 18 men) and proposed to add CCS hyperintensity as a new major radiologic criterion for FXTAS. Subsequently, Renaud *et al*.^71^ confirmed the finding from Apartis *et al*. and further reported that the combination of CCS and MCP hyperintensity was more common in FXTAS than in other neurodegenerative disorders, e.g. multiple system atrophy of the cerebellar type, Parkinson’s disease, Alzheimer’s disease. A third study addressing the prevalence of CCS in FXTAS compared 15 men and 7 women with FXTAS to 15 male and 7 female controls.^72^ This study showed that the CCS sign occurred more frequently in men with FXTAS compared with controls but not in women with FXTAS. Compared with the MCP sign, the CCS sign showed higher sensitivity but lower specificity in both men and women with FXTAS. Schneider *et al*.^57^ found that the white matter hyperintensity of the CCS was seen in 34 of 53 women with FXTAS and in only 2 of 55 age-matched control women and was the most common MRI abnormality in females with FXTAS.

This suggests that the diagnostic criteria for FXTAS may need to be altered in females compared to males, particularly for early cases. Indeed, the diagnostic criteria for FXTAS were determined with the study of males only.^75^ Subsequent criteria for the diagnosis of FXTAS were modified^76^ to include the involvement of the splenium, which is seen in the majority of females with FXTAS.^57,70^ Hall *et al*.^76^ also added the feature of neuropathy, which occurs in the majority of FXTAS patients, but it is a common finding in the elderly with many causes, so it is just a minor criterion for FXTAS. Although the diagnostic criteria have been modified somewhat for females, there may be subsequent changes in the early stages of FXTAS as further follow-up studies are able to separate the psychiatric illness in females seen earlier here from more significant neuropathology associated with extended features of FXTAS over time.^31,77^ As new treatments for FXTAS develop, it is hoped that the earlier the diagnosis is established, the more likely the treatment will be effective.

### Limitations and future work

This study has several limitations. In the subtype clustering analysis, it was challenging to concisely summarize the differences between subtypes, especially given the relatively small sample sizes per subtype and the correspondingly large amounts of uncertainty in their event orderings (Figs S14 and S15). Larger sample sizes would enable us to precisely estimate models with more stratifying variables. In particular, the current dataset consists of 60% males and 40% females among premutation carriers (Fig. S1). The ongoing recruitment for the GP study focuses on recruiting more females, which will help add precision to models for females and rare events in the future. We had substantial amounts of missing data. As described in the methods (Imputation of missing data), we used the marginal distribution of the observed data for each symptom among cases to impute the underlying values of these missing data. The missing data could contribute to uncertainty in the results. Our data contained both continuous and ordinal variables. In order to apply the Ordinal SuStaIn algorithm, we categorized some symptoms that were originally measured as continuous values. In doing so, we likely sacrificed some granular information. There are other variations of the SuStaIn algorithm, such as *z*-score SuStaIn,^20^ which are designed to be used with only continuous measurements. Further development is warranted to combine Ordinal SuStaIn and *z*-score SuStaIn to fit an event-based model with both continuous and ordinal data.

Our data come from the GP and TRAX studies, which are longitudinal cohort studies with infrequent follow-up visits. These studies do not collect precise information about timing of symptom onsets, particularly for historical symptoms, so it is not feasible to use the time-to-event data to confirm the results from the Ordinal SuStaIn analysis. In our analysis approach, the event onsets are not modelled as a function of participant age; instead, each event’s onset timing is only modelled relative to the other events using control participant information as reference. Therefore, the sequence differences between the subtypes are all in relative terms: if one event moves earlier in the sequence when comparing one subtype to another, other events are pushed later in the sequence, even if only one event timing changes relative to age. Additional research including longitudinal data collecting precise ages of onset would be needed to verify our findings.

Longitudinal data with more frequent follow-up visits would also enable us to answer questions about patients’ responses to receiving FXTAS diagnoses; in particular, it would be interesting to observe whether anxiety and depressive symptoms increase close or shortly following the onset of FXTAS. So far, our studies show that anxiety and depressive disorders worsen with age and particularly before the onset of FXTAS.^58^

The data used in this analysis came from patients and controls who participated in longitudinal studies at the UC Davis MIND Institute and may not be directly generalizable to other diverse populations. The participants predominantly reported their primary race/ethnicity as ‘White’, and we did not have data from enough patients with other diverse racial/ethnic identities to accurately estimate stratified models by race/ethnicity. A larger study including a more diverse set of participants is warranted to support analyses with increased external validity.

The permutation test approach for comparing the stratified models between subpopulations is a widely used inference technique, a desired method particularly when the true null distribution is unknown. The permutation test approach is based on the empirical sampling distribution of its own data to calculate a test statistic and its respective *P*-value. Its performance in this application could be validated with a large study where the empirical distribution of data tends to reflect the true distribution more closely.

When we compared between 55–99 versus 100–200 CGG repeats in males and females combined, no significant difference was found (*P* = 0.16) (Fig. S6). The finding of only minimal differences between 55–99 versus 100–200 CGG repeats is surprising, since several studies have found that the higher the CGG repeats, the earlier the onset and the faster the progression of FXTAS.^14,17,78^ Perhaps the cut-off of 100 is too high for this distinction to be made, and thus searching for an appropriate cut-off of CGG repeats is warranted to determine if a specific threshold of CGG repeat size triggers the progression of FXTAS.

## Supplementary Material

fcaf483_Supplementary_Data

## Data Availability

Data sharing is not applicable to this article as no new data were created or analysed in this study. The de-identified data used in this analysis may be provided upon request from the principal investigators of the two cohorts. The code used to perform this analysis is available through a repository at https://github.com/UCD-IDDRC/fxtas.
