# Supplementary material for: Progression of fragile X-associated tremor/ataxia syndrome revealed by subtype and stage inference
Source: Brain Commun. 2025 Dec 10;8(1):fcaf483. doi: 10.1093/braincomms/fcaf483 (PMC12776014; doi:10.1093/braincomms/fcaf483)
Supplement: fcaf483_Supplementary_Data [file fcaf483_supplementary_data.pdf]

Supplementary material

Summary data tables

The following supplementary tables 1 - 13 summarize the distributions of the symptoms that we analyzed in this paper, stratified by sex and CGG level.

Supplementary Table 1: **FXTAS Stage by CGG level and sex**

	Male			Female		
	**CGG <55 (N=37)**	**CGG 55-99 (N=116)**	**CGG 100-199 (N=36)**	**CGG <55 (N=7)**	**CGG 55-99 (N=76)**	**CGG 100-199 (N=25)**
**FXTAS stage**						
0	36 (100%)	25 (22.3%)	5 (14.3%)	3 (100%)	24 (40.0%)	6 (28.6%)
1	0 (0%)	23 (20.5%)	5 (14.3%)	0 (0%)	5 (8.3%)	1 (4.8%)
2	0 (0%)	26 (23.2%)	2 (5.7%)	0 (0%)	10 (16.7%)	5 (23.8%)
3	0 (0%)	28 (25.0%)	11 (31.4%)	0 (0%)	13 (21.7%)	9 (42.9%)
4	0 (0%)	8 (7.1%)	8 (22.9%)	0 (0%)	5 (8.3%)	0 (0%)
5	0 (0%)	2 (1.8%)	4 (11.4%)	0 (0%)	3 (5.0%)	0 (0%)
Missing	1 (2.7%)	4 (3.4%)	1 (2.8%)	4 (57.1%)	16 (21.1%)	4 (16.0%)

Supplementary Table 2: **Tremors by CGG level and sex**

	Male			Female		
	**CGG <55 (N=37)**	**CGG 55-99 (N=116)**	**CGG 100-199 (N=36)**	**CGG <55 (N=7)**	**CGG 55-99 (N=76)**	**CGG 100-199 (N=25)**
**Intention tremor**						
No	28 (84.8%)	41 (40.6%)	8 (24.2%)	3 (100%)	24 (40.0%)	6 (30.0%)
Yes	5 (15.2%)	60 (59.4%)	25 (75.8%)	0 (0%)	36 (60.0%)	14 (70.0%)
Missing	4 (10.8%)	15 (12.9%)	3 (8.3%)	4 (57.1%)	16 (21.1%)	5 (20.0%)
**Resting tremor**						
No	32 (94.1%)	79 (80.6%)	26 (74.3%)	3 (100%)	48 (80.0%)	14 (73.7%)
Yes	2 (5.9%)	19 (19.4%)	9 (25.7%)	0 (0%)	12 (20.0%)	5 (26.3%)
Missing	3 (8.1%)	18 (15.5%)	1 (2.8%)	4 (57.1%)	16 (21.1%)	6 (24.0%)
**Postural tremor**						
No	27 (81.8%)	56 (58.3%)	18 (56.3%)	3 (100%)	31 (52.5%)	8 (44.4%)
Yes	6 (18.2%)	40 (41.7%)	14 (43.8%)	0 (0%)	28 (47.5%)	10 (55.6%)
Missing	4 (10.8%)	20 (17.2%)	4 (11.1%)	4 (57.1%)	17 (22.4%)	7 (28.0%)
**Intermittent tremor**						
No	27 (93.1%)	45 (61.6%)	23 (85.2%)	3 (100%)	37 (69.8%)	10 (71.4%)
Yes	2 (6.9%)	28 (38.4%)	4 (14.8%)	0 (0%)	16 (30.2%)	4 (28.6%)
Missing	8 (21.6%)	43 (37.1%)	9 (25.0%)	4 (57.1%)	23 (30.3%)	11 (44.0%)

Supplementary Table 3: **Head tremors by CGG level and sex**

	Male			Female		
	**CGG <55 (N=37)**	**CGG 55-99 (N=116)**	**CGG 100-199 (N=36)**	**CGG <55 (N=7)**	**CGG 55-99 (N=76)**	**CGG 100-199 (N=25)**
**Head tremor**						
No	5 (100%)	36 (81.8%)	8 (57.1%)	0 (NA%)	16 (55.2%)	7 (53.8%)
Yes	0 (0%)	8 (18.2%)	6 (42.9%)	0 (NA%)	13 (44.8%)	6 (46.2%)
Missing	32 (86.5%)	72 (62.1%)	22 (61.1%)	7 (100%)	47 (61.8%)	12 (48.0%)

Supplementary Table 4: **Ataxia by CGG status**

	Male			Female		
	**CGG <55 (N=37)**	**CGG 55-99 (N=116)**	**CGG 100-199 (N=36)**	**CGG <55 (N=7)**	**CGG 55-99 (N=76)**	**CGG 100-199 (N=25)**
**Tandem walk**						
Normal	5 (100%)	8 (21.1%)	0 (0%)	3 (100%)	26 (49.1%)	8 (42.1%)
Abnormal (<10 steps)	0 (0%)	13 (34.2%)	5 (23.8%)	0 (0%)	13 (24.5%)	4 (21.1%)
Unable (Absent)	0 (0%)	17 (44.7%)	16 (76.2%)	0 (0%)	14 (26.4%)	7 (36.8%)
Missing	32 (86.5%)	78 (67.2%)	15 (41.7%)	4 (57.1%)	23 (30.3%)	6 (24.0%)
**Gait ataxia**						
No	31 (91.2%)	55 (51.4%)	9 (25.7%)	3 (100%)	29 (46.0%)	4 (19.0%)
Yes	3 (8.8%)	52 (48.6%)	26 (74.3%)	0 (0%)	34 (54.0%)	17 (81.0%)
Missing	3 (8.1%)	9 (7.8%)	1 (2.8%)	4 (57.1%)	13 (17.1%)	4 (16.0%)
**Ataxia severity**						
0	32 (94.1%)	70 (66.0%)	18 (51.4%)	3 (100%)	43 (69.4%)	12 (57.1%)
1	2 (5.9%)	21 (19.8%)	3 (8.6%)	0 (0%)	10 (16.1%)	3 (14.3%)
2	0 (0%)	9 (8.5%)	2 (5.7%)	0 (0%)	6 (9.7%)	2 (9.5%)
3	0 (0%)	5 (4.7%)	7 (20.0%)	0 (0%)	0 (0%)	4 (19.0%)
4	0 (0%)	1 (0.9%)	5 (14.3%)	0 (0%)	3 (4.8%)	0 (0%)
Missing	3 (8.1%)	10 (8.6%)	1 (2.8%)	4 (57.1%)	14 (18.4%)	4 (16.0%)

Supplementary Table 5: **Parkinson's disease and parkinsonian features, by CGG status**

	Male			Female		
	**CGG <55 (N=37)**	**CGG 55-99 (N=116)**	**CGG 100-199 (N=36)**	**CGG <55 (N=7)**	**CGG 55-99 (N=76)**	**CGG 100-199 (N=25)**
**Parkinson's disease**						
No	5 (100%)	13 (81.3%)	7 (77.8%)	0 (NA%)	10 (76.9%)	5 (83.3%)
Yes	0 (0%)	3 (18.8%)	2 (22.2%)	0 (NA%)	3 (23.1%)	1 (16.7%)
Missing	32 (86.5%)	100 (86.2%)	27 (75.0%)	7 (100%)	63 (82.9%)	19 (76.0%)
**Parkinsonian features**						
No	30 (100%)	62 (77.5%)	15 (51.7%)	2 (100%)	44 (84.6%)	16 (84.2%)
Yes	0 (0%)	18 (22.5%)	14 (48.3%)	0 (0%)	8 (15.4%)	3 (15.8%)
Missing	7 (18.9%)	36 (31.0%)	7 (19.4%)	5 (71.4%)	24 (31.6%)	6 (24.0%)

Supplementary Table 6: **Prevalence of parkinsonian features by FXTAS Stage among premutation carriers** (excluding observations with missing data for parkinsonian features).

		parkinsonian features	
**Characteristic**	**Overall N = 180**	**No N = 137**	**Yes N = 43**
FXTAS Stage			
0-2	107 (100%)	99 (93%)	8 (7.5%)
3+	71 (100%)	37 (52%)	34 (48%)
Missing	2	1	1

Supplementary Table 7: **Behavior Dyscontrol Scale - Second Edition (BDS-2)**

	Male			Female		
	**CGG <55 (N=37)**	**CGG 55-99 (N=116)**	**CGG 100-199 (N=36)**	**CGG <55 (N=7)**	**CGG 55-99 (N=76)**	**CGG 100-199 (N=25)**
**BDS-2 total score**						
Mean (SD)	22.3 (2.9)	20.7 (4.8)	17.4 (5.9)	23.3 (2.9)	21.3 (4.2)	21.4 (2.7)
Median (Min, Max)	23.0 (15, 27)	22.0 (2, 27)	19.0 (1, 26)	24.5 (19, 25)	22.0 (4, 26)	22.0 (17, 26)
Missing	1 (2.7%)	17 (14.7%)	5 (13.9%)	3 (42.9%)	24 (31.6%)	4 (16.0%)
**BDS-2 total score**						
≥ 20	30 (81.1%)	69 (59.5%)	14 (38.9%)	3 (42.9%)	42 (55.3%)	15 (60.0%)
< 20	6 (16.2%)	30 (25.9%)	17 (47.2%)	1 (14.3%)	10 (13.2%)	6 (24.0%)
Missing	1 (2.7%)	17 (14.7%)	5 (13.9%)	3 (42.9%)	24 (31.6%)	4 (16.0%)

Supplementary Table 8: **Mini-Mental State Examination (MMSE)**

	Male			Female		
	**CGG <55 (N=37)**	**CGG 55-99 (N=116)**	**CGG 100-199 (N=36)**	**CGG <55 (N=7)**	**CGG 55-99 (N=76)**	**CGG 100-199 (N=25)**
**MMSE total score**						
Mean (SD)	29.7 (0.6)	27.4 (4.3)	26.5 (3.4)	29.8 (0.5)	28.6 (2.4)	28.8 (1.5)
Median (Min, Max)	30.0 (29, 30)	29.0 (13, 30)	27.0 (16, 30)	30.0 (29, 30)	29.0 (16, 30)	29.0 (25, 30)
Missing	34 (91.9%)	73 (62.9%)	17 (47.2%)	3 (42.9%)	27 (35.5%)	3 (12.0%)
**MMSE total score**						
Normal (26-30)	3 (8.1%)	36 (31.0%)	13 (36.1%)	4 (57.1%)	46 (60.5%)	21 (84.0%)
Mild impairment (20-25)	0 (0%)	3 (2.6%)	5 (13.9%)	0 (0%)	2 (2.6%)	1 (4.0%)
Moderate impairment (10-19)	0 (0%)	4 (3.4%)	1 (2.8%)	0 (0%)	1 (1.3%)	0 (0%)
Missing	34 (91.9%)	73 (62.9%)	17 (47.2%)	3 (42.9%)	27 (35.5%)	3 (12.0%)

Supplementary Table 9: **Structured Clinical Interview for DSM Disorders (SCID)**

	Male			Female		
	**CGG <55 (N=37)**	**CGG 55-99 (N=116)**	**CGG 100-199 (N=36)**	**CGG <55 (N=7)**	**CGG 55-99 (N=76)**	**CGG 100-199 (N=25)**
**Mood disorders**						
Absent	19 (51.4%)	52 (44.8%)	14 (38.9%)	1 (14.3%)	17 (22.4%)	6 (24.0%)
Sub-Threshold	2 (5.4%)	7 (6.0%)	3 (8.3%)	0 (0%)	2 (2.6%)	0 (0%)
Threshold	11 (29.7%)	26 (22.4%)	9 (25.0%)	2 (28.6%)	32 (42.1%)	12 (48.0%)
Missing	5 (13.5%)	31 (26.7%)	10 (27.8%)	4 (57.1%)	25 (32.9%)	7 (28.0%)
**Substance use disorders**						
Absent	26 (70.3%)	59 (50.9%)	17 (47.2%)	3 (42.9%)	43 (56.6%)	14 (56.0%)
Sub-Threshold	2 (5.4%)	3 (2.6%)	0 (0%)	0 (0%)	3 (3.9%)	0 (0%)
Threshold	3 (8.1%)	23 (19.8%)	9 (25.0%)	0 (0%)	5 (6.6%)	4 (16.0%)
Missing	6 (16.2%)	31 (26.7%)	10 (27.8%)	4 (57.1%)	25 (32.9%)	7 (28.0%)
**Anxiety disorders**						
Absent	12 (32.4%)	31 (26.7%)	12 (33.3%)	0 (0%)	7 (9.2%)	2 (8.0%)
Sub-Threshold	8 (21.6%)	16 (13.8%)	1 (2.8%)	0 (0%)	7 (9.2%)	1 (4.0%)
Threshold	12 (32.4%)	38 (32.8%)	13 (36.1%)	3 (42.9%)	37 (48.7%)	15 (60.0%)
Missing	5 (13.5%)	31 (26.7%)	10 (27.8%)	4 (57.1%)	25 (32.9%)	7 (28.0%)
**Somatoform disorders**						
Absent	30 (81.1%)	84 (72.4%)	25 (69.4%)	2 (28.6%)	43 (56.6%)	15 (60.0%)
Sub-Threshold	1 (2.7%)	0 (0%)	0 (0%)	0 (0%)	2 (2.6%)	1 (4.0%)
Threshold	0 (0%)	1 (0.9%)	1 (2.8%)	1 (14.3%)	5 (6.6%)	2 (8.0%)
Missing	6 (16.2%)	31 (26.7%)	10 (27.8%)	4 (57.1%)	26 (34.2%)	7 (28.0%)

Supplementary Table 10: **Variables contributing to the "Mood Disorders" composite variable, by Sex and CGG level**

	Male CGG <55 (N=37)	Male CGG 55-99 (N=116)	Male CGG 100-199 (N=36)	Female CGG <55 (N=7)	Female CGG 55-99 (N=76)	Female CGG 100-199 (N=25)
**Bipolar I Disorder (MD01), Lifetime**						
Absent	32 (100%)	85 (100%)	26 (100%)	3 (100%)	50 (98.0%)	17 (94.4%)
Threshold	0 (0%)	0 (0%)	0 (0%)	0 (0%)	1 (2.0%)	1 (5.6%)
Missing	5 (13.5%)	31 (26.7%)	10 (27.8%)	4 (57.1%)	25 (32.9%)	7 (28.0%)
**Bipolar II Disorder (MD02), Lifetime**						
Absent	31 (96.9%)	83 (98.8%)	26 (100%)	3 (100%)	50 (98.0%)	17 (100%)
Threshold	1 (3.1%)	1 (1.2%)	0 (0%)	0 (0%)	1 (2.0%)	0 (0%)
Missing	5 (13.5%)	32 (27.6%)	10 (27.8%)	4 (57.1%)	25 (32.9%)	8 (32.0%)
**Other Bipolar Disorder (MD03), Lifetime**						
Absent	32 (100%)	84 (100%)	26 (100%)	3 (100%)	50 (98.0%)	17 (94.4%)
Sub-Threshold	0 (0%)	0 (0%)	0 (0%)	0 (0%)	1 (2.0%)	0 (0%)
Threshold	0 (0%)	0 (0%)	0 (0%)	0 (0%)	0 (0%)	1 (5.6%)
Missing	5 (13.5%)	32 (27.6%)	10 (27.8%)	4 (57.1%)	25 (32.9%)	7 (28.0%)
**Major Depressive Disorder (MD04), Lifetime**						
Absent	24 (75.0%)	58 (68.2%)	17 (65.4%)	1 (33.3%)	22 (43.1%)	10 (55.6%)
Sub-Threshold	2 (6.3%)	8 (9.4%)	3 (11.5%)	0 (0%)	3 (5.9%)	0 (0%)
Threshold	6 (18.8%)	19 (22.4%)	6 (23.1%)	2 (66.7%)	26 (51.0%)	8 (44.4%)
Missing	5 (13.5%)	31 (26.7%)	10 (27.8%)	4 (57.1%)	25 (32.9%)	7 (28.0%)
**Dysthymic Disorder (MD05), Lifetime**						
Absent	29 (93.5%)	80 (95.2%)	24 (92.3%)	3 (100%)	49 (96.1%)	16 (88.9%)
Sub-Threshold	0 (0%)	0 (0%)	0 (0%)	0 (0%)	1 (2.0%)	0 (0%)
Threshold	2 (6.5%)	4 (4.8%)	2 (7.7%)	0 (0%)	1 (2.0%)	2 (11.1%)
Missing	6 (16.2%)	32 (27.6%)	10 (27.8%)	4 (57.1%)	25 (32.9%)	7 (28.0%)
**Depressive Disorder NOS (MD06), Lifetime**						
Absent	28 (87.5%)	80 (94.1%)	22 (91.7%)	3 (100%)	48 (94.1%)	16 (88.9%)
Threshold	4 (12.5%)	5 (5.9%)	2 (8.3%)	0 (0%)	3 (5.9%)	2 (11.1%)
Missing	5 (13.5%)	31 (26.7%)	12 (33.3%)	4 (57.1%)	25 (32.9%)	7 (28.0%)
**Mood Disorder Due to GMC (MD07), Lifetime**						
Absent	32 (100%)	83 (97.6%)	26 (100%)	3 (100%)	50 (98.0%)	18 (100%)
Threshold	0 (0%)	2 (2.4%)	0 (0%)	0 (0%)	1 (2.0%)	0 (0%)
Missing	5 (13.5%)	31 (26.7%)	10 (27.8%)	4 (57.1%)	25 (32.9%)	7 (28.0%)
**Substance-Induced Mood Dis. (MD08), Lifetime**						
Absent	32 (100%)	84 (98.8%)	26 (100%)	3 (100%)	51 (100%)	18 (100%)
Threshold	0 (0%)	1 (1.2%)	0 (0%)	0 (0%)	0 (0%)	0 (0%)
Missing	5 (13.5%)	31 (26.7%)	10 (27.8%)	4 (57.1%)	25 (32.9%)	7 (28.0%)

Supplementary Table 11: **MRI variables by Sex and CGG level**

	Male			Female		
	CGG <55 (N=37)	CGG 55-99 (N=116)	CGG 100-199 (N=36)	CGG <55 (N=7)	CGG 55-99 (N=76)	CGG 100-199 (N=25)
**MRI: Cerebellar**						
None	0 (NA%)	28 (43.1%)	1 (5.6%)	1 (100%)	9 (56.3%)	3 (33.3%)
Mild	0 (NA%)	26 (40.0%)	9 (50.0%)	0 (0%)	6 (37.5%)	6 (66.7%)
Moderate	0 (NA%)	7 (10.8%)	4 (22.2%)	0 (0%)	1 (6.3%)	0 (0%)
Severe	0 (NA%)	4 (6.2%)	4 (22.2%)	0 (0%)	0 (0%)	0 (0%)
Missing	37 (100%)	51 (44.0%)	18 (50.0%)	6 (85.7%)	60 (78.9%)	16 (64.0%)
**MRI: Cerebral**						
None	0 (NA%)	15 (21.7%)	1 (5.6%)	1 (100%)	0 (0%)	1 (11.1%)
Mild	0 (NA%)	35 (50.7%)	6 (33.3%)	0 (0%)	12 (70.6%)	5 (55.6%)
Moderate	0 (NA%)	15 (21.7%)	7 (38.9%)	0 (0%)	5 (29.4%)	3 (33.3%)
Severe	0 (NA%)	4 (5.8%)	4 (22.2%)	0 (0%)	0 (0%)	0 (0%)
Missing	37 (100%)	47 (40.5%)	18 (50.0%)	6 (85.7%)	59 (77.6%)	16 (64.0%)
**MCP white matter hyperintensity**						
None	0 (NA%)	47 (67.1%)	7 (36.8%)	1 (100%)	17 (100%)	9 (100%)
Mild	0 (NA%)	9 (12.9%)	3 (15.8%)	0 (0%)	0 (0%)	0 (0%)
Moderate	0 (NA%)	10 (14.3%)	7 (36.8%)	0 (0%)	0 (0%)	0 (0%)
Severe	0 (NA%)	4 (5.7%)	2 (10.5%)	0 (0%)	0 (0%)	0 (0%)
Missing	37 (100%)	46 (39.7%)	17 (47.2%)	6 (85.7%)	59 (77.6%)	16 (64.0%)
**Splenium white matter hyperintensity**						
None	0 (NA%)	21 (39.6%)	1 (7.7%)	1 (100%)	7 (41.2%)	1 (11.1%)
Mild	0 (NA%)	18 (34.0%)	1 (7.7%)	0 (0%)	6 (35.3%)	5 (55.6%)
Moderate	0 (NA%)	8 (15.1%)	6 (46.2%)	0 (0%)	3 (17.6%)	3 (33.3%)
Severe	0 (NA%)	6 (11.3%)	5 (38.5%)	0 (0%)	1 (5.9%)	0 (0%)
Missing	37 (100%)	63 (54.3%)	23 (63.9%)	6 (85.7%)	59 (77.6%)	16 (64.0%)
**Genu white matter hyperintensity**						
No	0 (NA%)	28 (58.3%)	2 (16.7%)	1 (100%)	9 (60.0%)	4 (50.0%)
Yes	0 (NA%)	20 (41.7%)	10 (83.3%)	0 (0%)	6 (40.0%)	4 (50.0%)
Missing	37 (100%)	68 (58.6%)	24 (66.7%)	6 (85.7%)	61 (80.3%)	17 (68.0%)
**Corpus callosum thickness**						
Normal	0 (NA%)	34 (65.4%)	1 (8.3%)	1 (100%)	13 (81.3%)	6 (66.7%)
Thin	0 (NA%)	18 (34.6%)	11 (91.7%)	0 (0%)	3 (18.8%)	3 (33.3%)
Missing	37 (100%)	64 (55.2%)	24 (66.7%)	6 (85.7%)	60 (78.9%)	16 (64.0%)

Supplementary Table 12: **Cambridge Neuropsychological Test Automated Battery (CANTAB)** We used categorization cutoffs taken from Talebi et al 2020.[1] SWM = Spatial Working Memory. PAL = Paired Associates Learning. RTI = Reaction Time.

	Male			Female		
	CGG <55 (N=37)	CGG 55-99 (N=116)	CGG 100-199 (N=36)	CGG <55 (N=7)	CGG 55-99 (N=76)	CGG 100-199 (N=25)
**SWM Between errors**						
Mean (SD)	31.3 (18.9)	27.5 (21.0)	32.8 (25.2)	12.5 (17.7)	15.8 (7.7)	17.3 (8.4)
Median (Min, Max)	26.0 (4.0, 74.0)	22.0 (0.0, 97.0)	25.0 (0.0, 81.0)	12.5 (0.0, 25.0)	16.5 (0.0, 27.0)	19.0 (5.0, 27.0)
Missing	14 (37.8%)	37 (31.9%)	15 (41.7%)	5 (71.4%)	64 (84.2%)	19 (76.0%)
**SWM between errors**						
≤ 26	12 (32.4%)	51 (44.0%)	13 (36.1%)	2 (28.6%)	11 (14.5%)	5 (20.0%)
> 26	11 (29.7%)	28 (24.1%)	8 (22.2%)	0 (0%)	1 (1.3%)	1 (4.0%)
Missing	14 (37.8%)	37 (31.9%)	15 (41.7%)	5 (71.4%)	64 (84.2%)	19 (76.0%)
**PAL Total errors (adjusted)**						
Mean (SD)	22.5 (17.5)	28.1 (22.0)	21.4 (14.0)	22.5 (27.6)	29.0 (21.2)	27.9 (19.4)
Median (Min, Max)	18.0 (2.0, 76.0)	23.0 (2.0, 100.0)	18.0 (2.0, 48.0)	22.5 (3.0, 42.0)	22.5 (7.0, 63.0)	15.0 (6.0, 54.0)
Missing	14 (37.8%)	38 (32.8%)	14 (38.9%)	5 (71.4%)	64 (84.2%)	18 (72.0%)
**PAL total errors**						
≤ 13	8 (21.6%)	26 (22.4%)	8 (22.2%)	1 (14.3%)	4 (5.3%)	1 (4.0%)
> 13	15 (40.5%)	52 (44.8%)	14 (38.9%)	1 (14.3%)	8 (10.5%)	6 (24.0%)
Missing	14 (37.8%)	38 (32.8%)	14 (38.9%)	5 (71.4%)	64 (84.2%)	18 (72.0%)
**RTI Five-choice movement time**						
Mean (SD)	241.2 (49.1)	343.9 (119.1)	416.5 (175.3)	308.5 (7.8)	358.3 (60.4)	286.0 (39.3)
Median (Min, Max)	234.5 (161.3, 343.5)	318.9 (138.8, 703.0)	401.2 (189.1, 950.0)	308.5 (303.0, 314.0)	368.5 (240.5, 459.0)	290.0 (234.5, 336.0)
Missing	14 (37.8%)	37 (31.9%)	14 (38.9%)	5 (71.4%)	63 (82.9%)	18 (72.0%)
**RTI five-choice movement time**						
≤ 368.57	23 (62.2%)	51 (44.0%)	10 (27.8%)	2 (28.6%)	7 (9.2%)	7 (28.0%)
> 368.57	0 (0%)	28 (24.1%)	12 (33.3%)	0 (0%)	6 (7.9%)	0 (0%)
Missing	14 (37.8%)	37 (31.9%)	14 (38.9%)	5 (71.4%)	63 (82.9%)	18 (72.0%)

Supplementary Table 13: **Thyroid and autoimmune diseases**

	Male			Female		
	CGG <55 (N=37)	CGG 55-99 (N=116)	CGG 100-199 (N=36)	CGG <55 (N=7)	CGG 55-99 (N=76)	CGG 100-199 (N=25)
**Hypothyroid**						
No	24 (100%)	58 (86.6%)	24 (82.8%)	2 (66.7%)	44 (77.2%)	15 (88.2%)
Yes	0 (0%)	9 (13.4%)	5 (17.2%)	1 (33.3%)	13 (22.8%)	2 (11.8%)
Missing	13 (35.1%)	49 (42.2%)	7 (19.4%)	4 (57.1%)	19 (25.0%)	8 (32.0%)
**Hyperthyroid**						
No	23 (95.8%)	65 (100%)	26 (96.3%)	3 (100%)	49 (96.1%)	16 (100%)
Yes	1 (4.2%)	0 (0%)	1 (3.7%)	0 (0%)	2 (3.9%)	0 (0%)
Missing	13 (35.1%)	51 (44.0%)	9 (25.0%)	4 (57.1%)	25 (32.9%)	9 (36.0%)
**Pulmonary fibrosis**						
No	5 (100%)	34 (100%)	17 (100%)	0 (NA%)	28 (100%)	13 (100%)
Missing	32 (86.5%)	82 (70.7%)	19 (52.8%)	7 (100%)	48 (63.2%)	12 (48.0%)
**Autoimmune diagnoses or symptoms**						
No	33 (97.1%)	89 (89.0%)	34 (97.1%)	3 (100%)	49 (77.8%)	17 (81.0%)
Yes	1 (2.9%)	11 (11.0%)	1 (2.9%)	0 (0%)	14 (22.2%)	4 (19.0%)
Missing	3 (8.1%)	16 (13.8%)	1 (2.8%)	4 (57.1%)	13 (17.1%)	4 (16.0%)

Estimates of event scoring accuracy

Supplementary Table 14 shows the estimated percentage of correctly scored individuals for each symptom,[2] estimated using the controls data.

Supplementary Table 14: **Percentages of controls at baseline levels, and corresponding estimates of probability of correct scoring.** The percentage of correctly scored individuals is estimated as the percentage of controls who were assessed as being at the reference level, or 95%, whichever was smaller.

Symptom	# controls with data	# at baseline	% at baseline	Est. Pr(correct)
head tremor	5	5	100%	95%
intention tremor	36	31	86.1%	86.1%
resting tremor	37	35	94.6%	94.6%
postural tremor	36	30	83.3%	83.3%
intermittent tremor	32	30	93.8%	93.8%
tandem walk	8	8	100%	95%
ataxia severity	37	35	94.6%	94.6%
FXTAS stage	39	39	100%	95%
parkinsonian features	32	32	100%	95%
Parkinson's disease	5	5	100%	95%
MCP white matter hyperintensity	1	1	100%	95%
MRI: Cerebellar	1	1	100%	95%
MRI: Cerebral	1	1	100%	95%
splenium white matter hyperintensity	1	1	100%	95%
genu white matter hyperintensity	1	1	100%	95%
corpus callosum thickness	1	1	100%	95%
MMSE total score	7	7	100%	95%
BDS-2 total score	40	33	82.5%	82.5%
mood disorders	35	22	62.9%	62.9%
substance use disorders	34	31	91.2%	91.2%
anxiety disorders	35	20	57.1%	57.1%
somatoform disorders	34	33	97.1%	95%
SWM between errors	25	14	56%	56%
PAL total errors	25	9	36%	36%
RTI five-choice movement time	25	25	100%	95%
hypothyroid	27	26	96.3%	95%
hyperthyroid	27	26	96.3%	95%
autoimmune diagnoses or symptoms	37	36	97.3%	95%

Composite variables

Autoimmune diagnoses or symptoms

Due to the infrequent nature of individual autoimmune diseases, a composite variable, “autoimmune diagnoses or symptoms”, was created for analysis: "Yes" if a patient had any of systemic lupus erythematosus, rheumatoid arthritis, multiple sclerosis, positive ANA (anti-nuclear antibody), Sjogren’s syndrome, or Raynaud’s syndrome; otherwise, "No" for no autoimmune diseases recorded.

MRI variables

The MRI variables are combined into composite variables taking the most severe score within a brain region.

- "MRI Cerebellar" was composited as the most severe score of Cerebellar atrophy, Cerebellar white matter (WM) hyperintensity, and Middle cerebellar peduncle (MCP) WM hyperintensity.

- "MRI Cerebral" was composited as the most severe score of Cerebral atrophy, Cerebral WM hyperintensity, Pons WM hyperintensity, Sub-insular WM hyperintensity, and Periventricular WM hyperintensity.

- Splenium WM hyperintensity, Genu WM hyperintensity, and Corpus Callosum thickness remained as separate fields, because they had been coded using incompatible Likert scales.

SCID composite variables

Similarly, we combined individual SCID disorders into composite variables, taking the highest level ("Absent", "Sub-Threshold", or "Threshold") among the constituent individual disorders.

- "SCID: mood disorders" combines: Bipolar I Disorder (MD01), Lifetime, Bipolar II Disorder (MD02), Lifetime, Other Bipolar Disorder (MD03), Lifetime, Major Depressive Disorder (MD04), Lifetime, Dysthymic Disorder (MD05), Lifetime, Depressive Disorder NOS (MD06), Lifetime, Mood Disorder Due to GMC (MD07), Lifetime, Substance-Induced Mood Dis. (MD08), Lifetime.

- "SCID: substance use disorders" combines Alcohol (SUD17), Lifetime, Sedative-Hypnotic-Anxiolytic (SUD18), Lifetime, Cannabis (SUD19), Lifetime, Stimulants (SUD20), Lifetime, Opioid (SUD21), Lifetime, Cocaine (SUD22), Lifetime, Hallucinogenics/ PCP (SUD23), Lifetime, Poly Drug (SUD24), Lifetime, Other (SUD25), Lifetime.

- "SCID: anxiety disorders" combines Panic Disorder (ANX26), Lifetime, Agoraphobia without Panic (ANX27), Lifetime, Social Phobia (ANX28), Lifetime,

Specific Phobia (ANX29), Lifetime, Obsessive Compulsive (ANX30), Lifetime, Posttraumatic Stress (ANX31), Lifetime, Generalized Anxiety (ANX32), Current Only, Anxiety Due to GMC (ANX33), Lifetime, Substance-Induced Anxiety (ANX34), Lifetime, Anxiety Disorder NOS (ANX35), Lifetime. Note that in the SCID-I/NP for DSM-IV, for generalized anxiety, only the current, not lifetime prevalence, is included.

- "SCID: somatoform disorders" combines Somatization Disorder (SOM36), Pain Disorder (SOM37), Undifferentiated Somatoform (SOM38), Body Dysmorphic (SOM40), Hypochondriasis (SOM39).

- "SCID: psychotic symptoms" consists of only Primary Psychotic Symptoms (PS01), Lifetime.

We then combined the "Absent" and "Sub-Threshold" levels in order to reduce the number of stages in the model, due to our limited sample size (as described in the main text, Symptoms of neurodegenerative events), and since very relatively participants had composite SCID variables at the sub-threshold levels (Supplementary Table 9).

Supplementary Table 10 shows contingency tables for the individual SCID items included in the "mood disorders" composite variable. Most participants with mood disorders had Major Depressive Disorder (MD04); a few had bipolar disorders (MD01, MD02, MD03).

Analyses stratified by sex

Supplementary Fig. 1: **Distribution of estimated disease stage, stratified by sex**. Red horizontal line indicates minimum sample size recommended for the Ordinal SuStaIn algorithm, three observations per stage.[2] Bar heights represent the number of premutation carriers (y-axis) clustered into each combination of subtype (panel) and stage (x-axis).

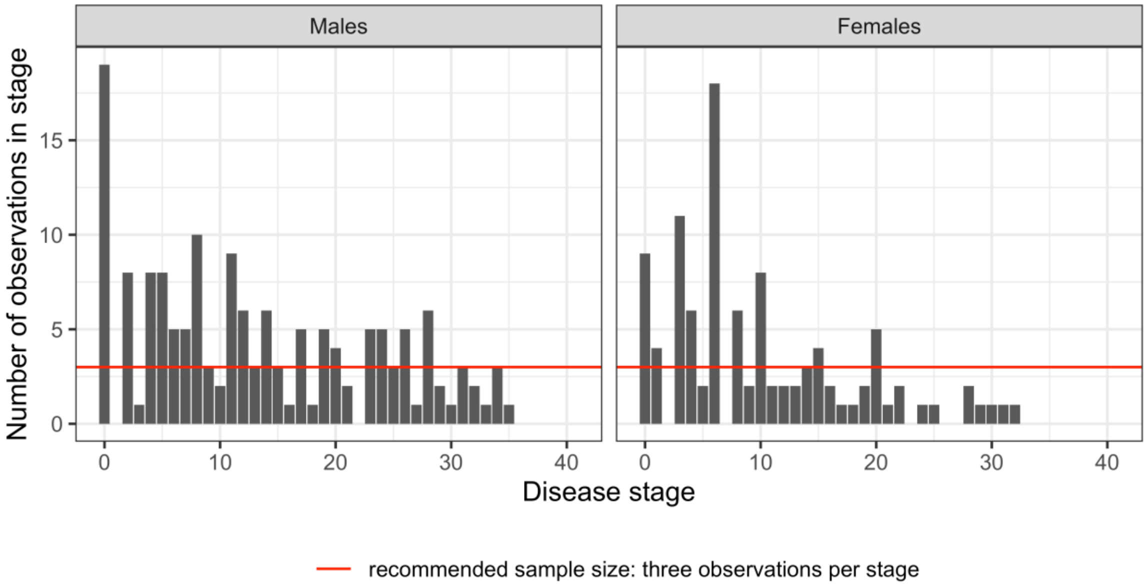

Comparing sexes stratified by CGG level

Supplementary Fig. 2: **Event sequences stratified by sex, for CGG repeats <100.** The heatmap colors (violet red, royal blue, cadet blue, khaki, forest green) indicate the ordinal levels of symptom progression (Table 2). Heatmap color gradient intensity represents the Bayesian posterior probability of the sequence position; the brighter the color, the more probable that the corresponding symptom event occurs in that position in the sequence. Label text colors indicate symptom categories (Table 2). Abbreviations: BDS-2 = Behavior Dyscontrol Scale - Second Edition; CANTAB = Cambridge Neuropsychological Test Automated Battery; FXTAS = Fragile X-associated tremor/ataxia syndrome; MCP = Middle cerebellar peduncle; MMSE = Mini-Mental State Exam; Mod. = Moderate; MRI = Magnetic Resonance Imaging; PAL = Paired Associates Learning; RTI = Reaction Time; SCID = Structured Clinical Interview for DSM Disorders; SWM = Spatial Working Memory. Permutation test statistic (log-likelihood): -4054.11; p-value = 0.022 (N = 192 premutation carriers).

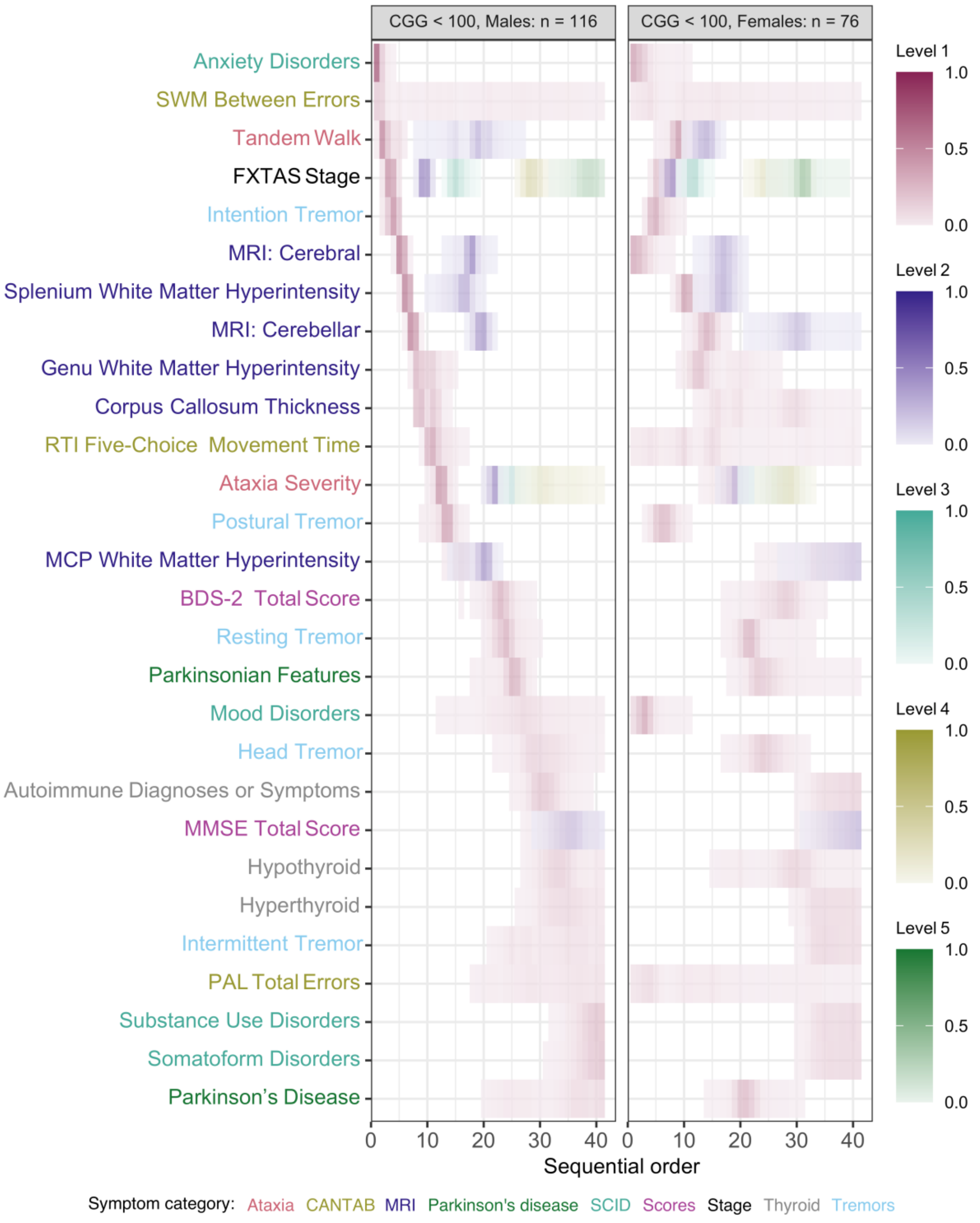

Supplementary Fig. 3: **Positional differences in event sequences by sex, for CGG repeats <100.** Red lines indicate symptoms that moved to later positions between the left-hand subgroup and the right-hand subgroup. Blue lines indicate symptoms that moved to earlier positions. Light gray lines indicate symptoms that did not change positions. Black lines indicate FXTAS stages. Line opacity levels indicate the number of positions changed (higher opacity represents more positions changed). Label text colors indicate symptom categories (Table 2). Yellow highlights indicate symptoms with clinically-significant positional differences between subgroups. Abbreviations: BDS-2 = Behavior Dyscontrol Scale - Second Edition; CANTAB = Cambridge Neuropsychological Test Automated Battery; FXTAS = Fragile X-associated tremor/ataxia syndrome; Hyp. = Hyperintensity; MCP = Middle cerebellar peduncle; MMSE = Mini-Mental State Exam; Mod. = Moderate; MRI = Magnetic Resonance Imaging; PAL = Paired Associates Learning; RTI = Reaction Time; SCID = Structured Clinical Interview for DSM Disorders; SWM = Spatial Working Memory; WM = White Matter. Permutation test statistic (log-likelihood): -4054.11; p-value = 0.022 (N = 192 premutation carriers).

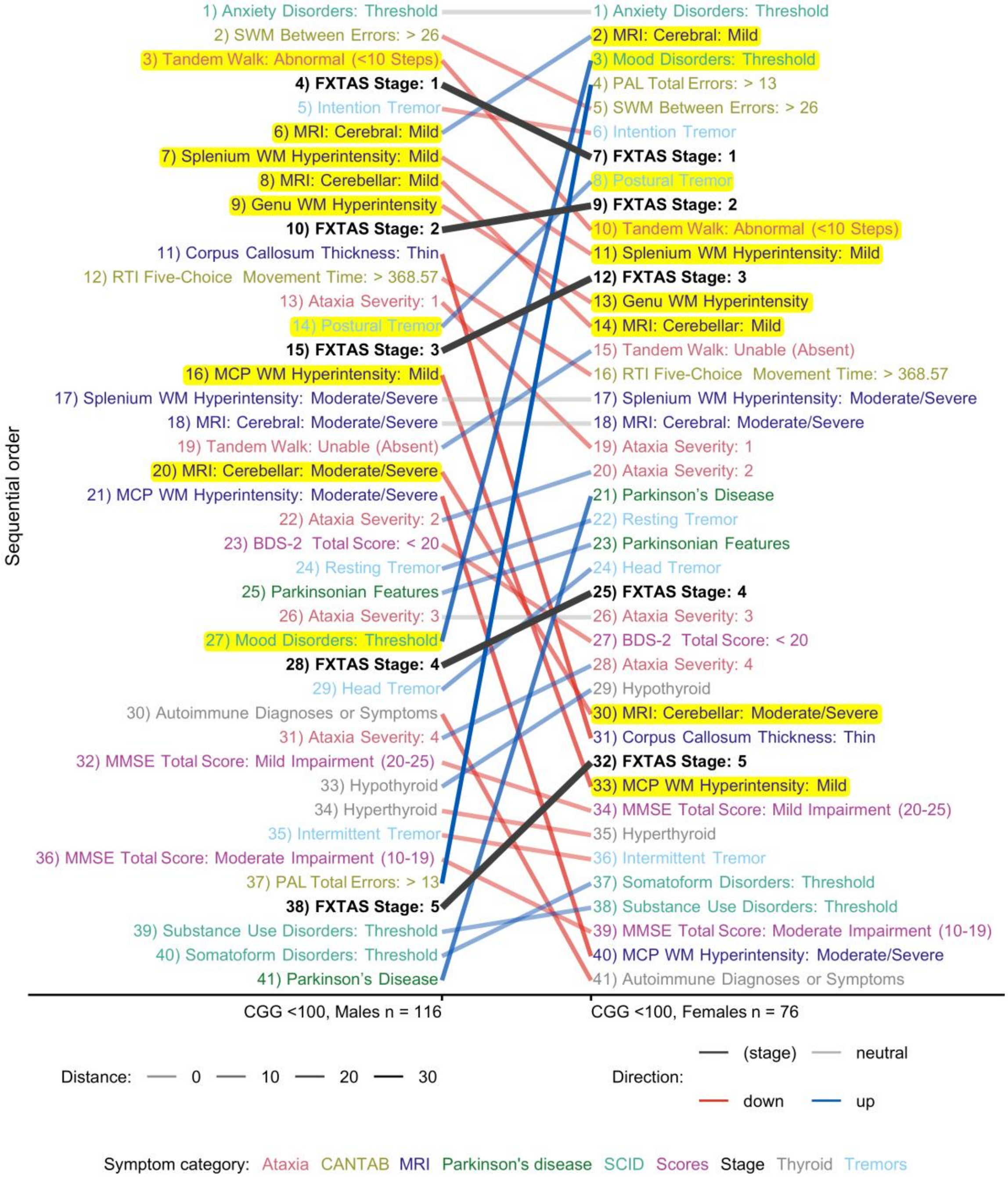

Supplementary Fig. 4: **Event sequences stratified by sex, for CGG repeats ≥100.** The heatmap colors (violet red, royal blue, cadet blue, khaki, forest green) indicate the ordinal levels of symptom progression (Table 2). Heatmap color gradient intensity represents the Bayesian posterior probability of the sequence position; the brighter the color, the more probable that the corresponding symptom event occurs in that position in the sequence. Label text colors indicate symptom categories (Table 2). Abbreviations: BDS-2 = Behavior Dyscontrol Scale - Second Edition; CANTAB = Cambridge Neuropsychological Test Automated Battery; FXTAS = Fragile X-associated tremor/ataxia syndrome; MCP = Middle cerebellar peduncle; MMSE = Mini-Mental State Exam; Mod. = Moderate; MRI = Magnetic Resonance Imaging; PAL = Paired Associates Learning; RTI = Reaction Time; SCID = Structured Clinical Interview for DSM Disorders; SWM = Spatial Working Memory. Permutation test statistic (log-likelihood): -1204.11; p-value = 0.178 (N = 61 premutation carriers).

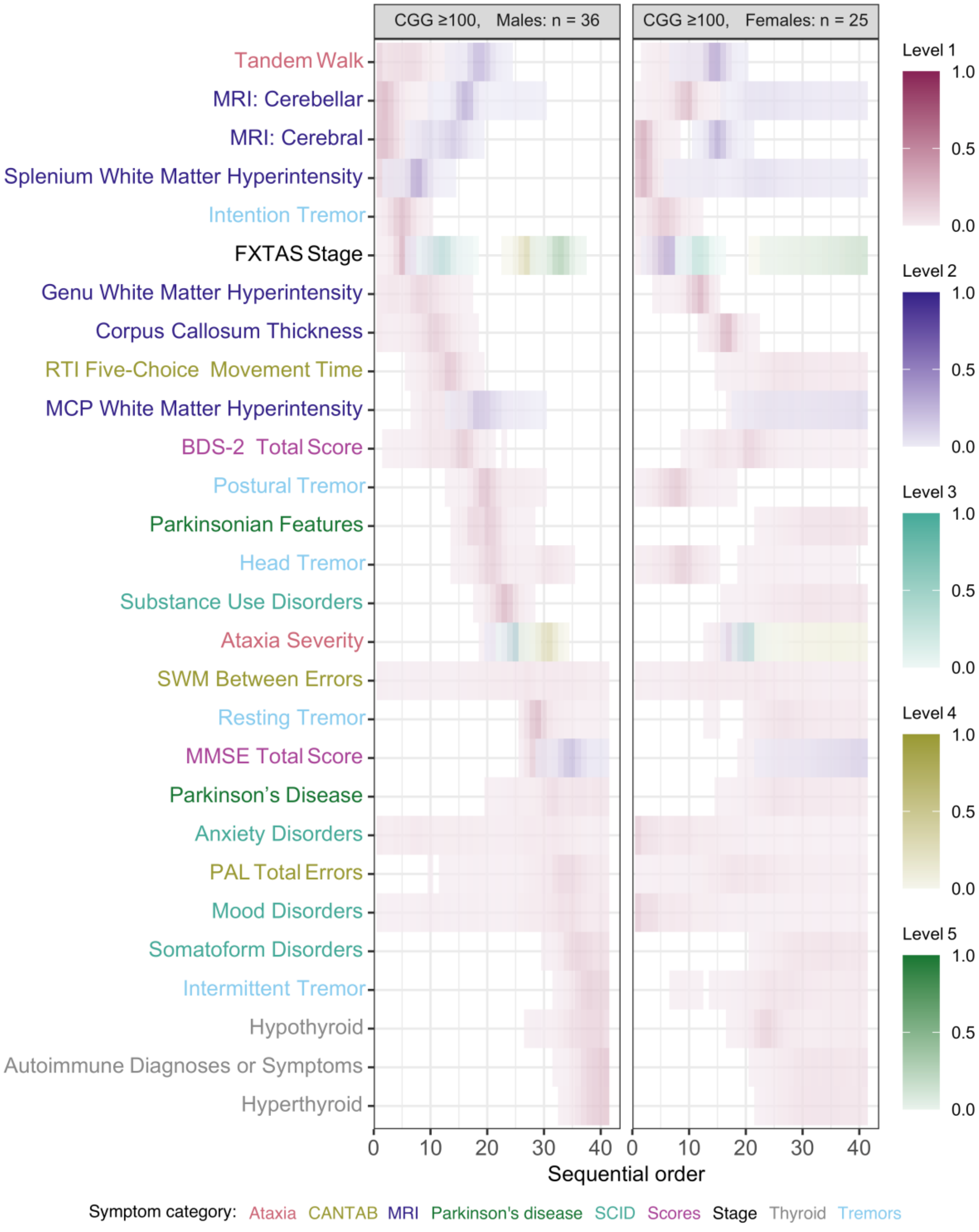

Supplementary Fig. 5: **Positional differences in event sequences by sex, for CGG repeats ≥100.** Red lines indicate symptoms that moved to later positions between the left-hand subgroup and the right-hand subgroup. Blue lines indicate symptoms that moved to earlier positions. Light gray lines indicate symptoms that did not change positions. Black lines indicate FXTAS stages. Line opacity levels indicate the number of positions changed (higher opacity represents more positions changed). Label text colors indicate symptom categories (Table 2). Yellow highlights indicate symptoms with clinically-significant positional differences between subgroups. Abbreviations: BDS-2 = Behavior Dyscontrol Scale - Second Edition; CANTAB = Cambridge Neuropsychological Test Automated Battery; FXTAS = Fragile X-associated tremor/ataxia syndrome; Hyp. = Hyperintensity; MCP = Middle cerebellar peduncle; MMSE = Mini-Mental State Exam; Mod. = Moderate; MRI = Magnetic Resonance Imaging; PAL = Paired Associates Learning; RTI = Reaction Time; SCID = Structured Clinical Interview for DSM Disorders; SWM = Spatial Working Memory; WM = White Matter. Permutation test statistic (log-likelihood): -1204.11; p-value = 0.178 (N = 61 premutation carriers).

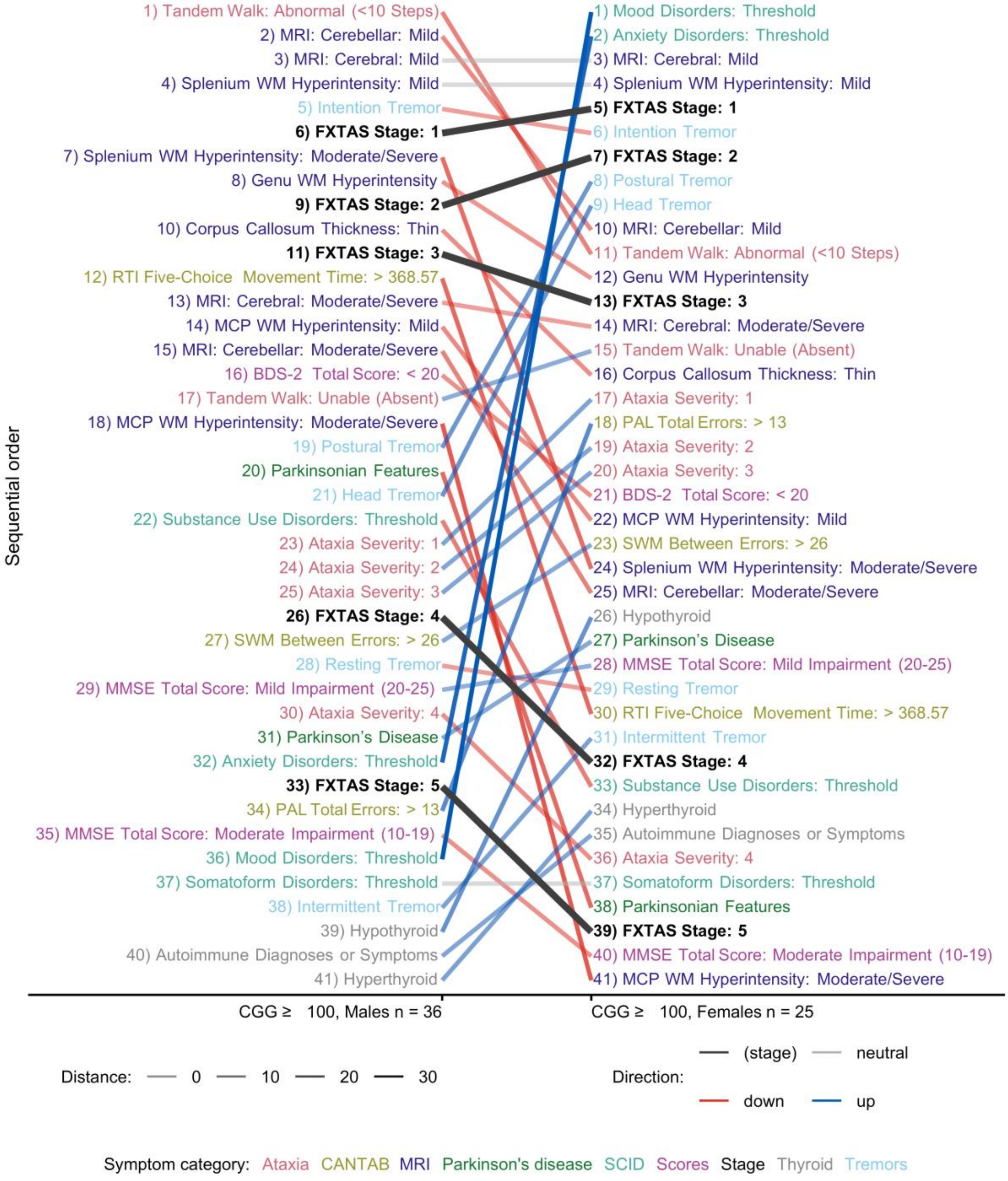

Analyses stratified by CGG repeats

Supplementary Fig. 6 shows the estimated sequences stratified by CGG repeat level. We did not find statistically significant evidence of a difference in event sequences between CGG < 100 and CGG ≥ 100 (p = 0.16), but some potential differences were noted (Supplementary Fig. 6 and Supplementary Fig. 7). SWM between errors occurred prior to Stage 1 in participants with CGG repeats <100, whereas it occurred between stage 4 and 5 in those with CGG repeats ≥ 100 (Supplementary Fig. 7). Postural, resting, head, and intermittent tremor occurred earlier in those with CGG repeats <100 compared to those with CGG repeats ≥100. Several psychiatric disorders (as measured by SCID) occurred later in the event sequence in participants with CGG repeats <100 compared to those with CGG repeats ≥100.

The finding of only minimal differences between 55-99 vs 100-199 CGG repeats is surprising, since several studies have found that the higher the CGG repeats, the earlier the onset and the faster the progression of FXTAS.[3–5] Perhaps the cut off of 100 is too high for this distinction to be made.

These differences were more evident among males than females (Supplementary material, Comparing CGG levels stratified by sex). When we fitted models for subgroup analyses by sex and CGG repeats level, the models for males showed the same patterns described above, except for head tremor (we found no difference in head tremor onset between CGG levels). Supplementary Fig. 8 shows the corresponding stratified models and Supplementary Fig. 9 compares the estimates of the sequences for these two subgroups.

We did not find statistically significant evidence of a difference between CGG <100 and CGG ≥100 among females (p = 0.73). Supplementary Fig. 10 shows the estimated stratified models and Supplementary Fig. 11 compares the estimates. Females had later onset of SWM Between Errors for CGG repeats ≥100 and later onset of intermittent tremors for CGG repeats <100, but did not show evidence of the other patterns observed for males and for the models without sex-stratification. Limited sample size in females for subgroup analyses was likely an issue; the available data included only 25 females with CGG ≥100.

Supplementary Fig. 6: **Event sequences stratified by CGG repeats (<100 vs 100+).** The heatmap colors (violet red, royal blue, cadet blue, khaki, forest green) indicate the ordinal levels of symptom progression (Table 2). Heatmap color gradient intensity represents the Bayesian posterior probability of the sequence position; the brighter the color, the more probable that the corresponding symptom event occurs in that position in the sequence. Label text colors indicate symptom categories (Table 2). Abbreviations: BDS-2 = Behavior Dyscontrol Scale - Second Edition; CANTAB = Cambridge Neuropsychological Test Automated Battery; FXTAS = Fragile X-associated tremor/ataxia syndrome; MCP = Middle cerebellar peduncle; MMSE = Mini-Mental State Exam; Mod. = Moderate; MRI = Magnetic Resonance Imaging; PAL = Paired Associates Learning; RTI = Reaction Time; SCID = Structured Clinical Interview for DSM Disorders; SWM = Spatial Working Memory. Permutation test statistic (log-likelihood): -5364.21; p-value = 0.160 (N = 253 premutation carriers).

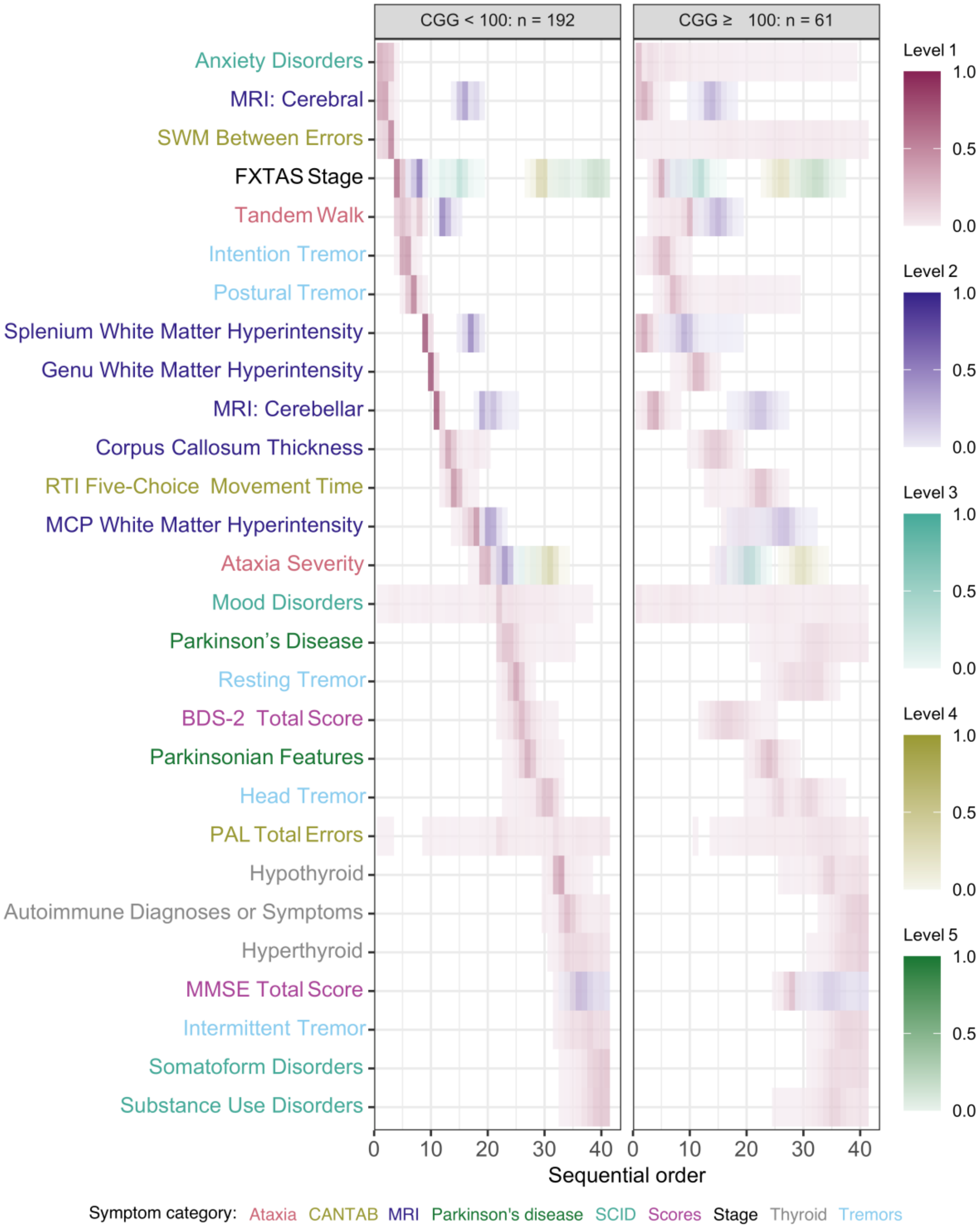

Supplementary Fig. 7: **Positional differences in event sequences stratified by CGG repeat sizes (<100 vs 100+).** Red lines indicate symptoms that moved to later positions between the left-hand subgroup and the right-hand subgroup. Blue lines indicate symptoms that moved to earlier positions. Light gray lines indicate symptoms that did not change positions. Black lines indicate FXTAS stages. Line opacity levels indicate the number of positions changed (higher opacity represents more positions changed). Label text colors indicate symptom categories (Table 2). Yellow highlights indicate symptoms with clinically-significant positional differences between subgroups. Abbreviations: BDS-2 = Behavior Dyscontrol Scale - Second Edition; CANTAB = Cambridge Neuropsychological Test Automated Battery; FXTAS = Fragile X-associated tremor/ataxia syndrome; Hyp. = Hyperintensity; MCP = Middle cerebellar peduncle; MMSE = Mini-Mental State Exam; Mod. = Moderate; MRI = Magnetic Resonance Imaging; PAL = Paired Associates Learning; RTI = Reaction Time; SCID = Structured Clinical Interview for DSM Disorders; SWM = Spatial Working Memory; WM = White Matter. Permutation test statistic (log-likelihood): -5364.21; p-value = 0.160 (N = 253 premutation carriers).

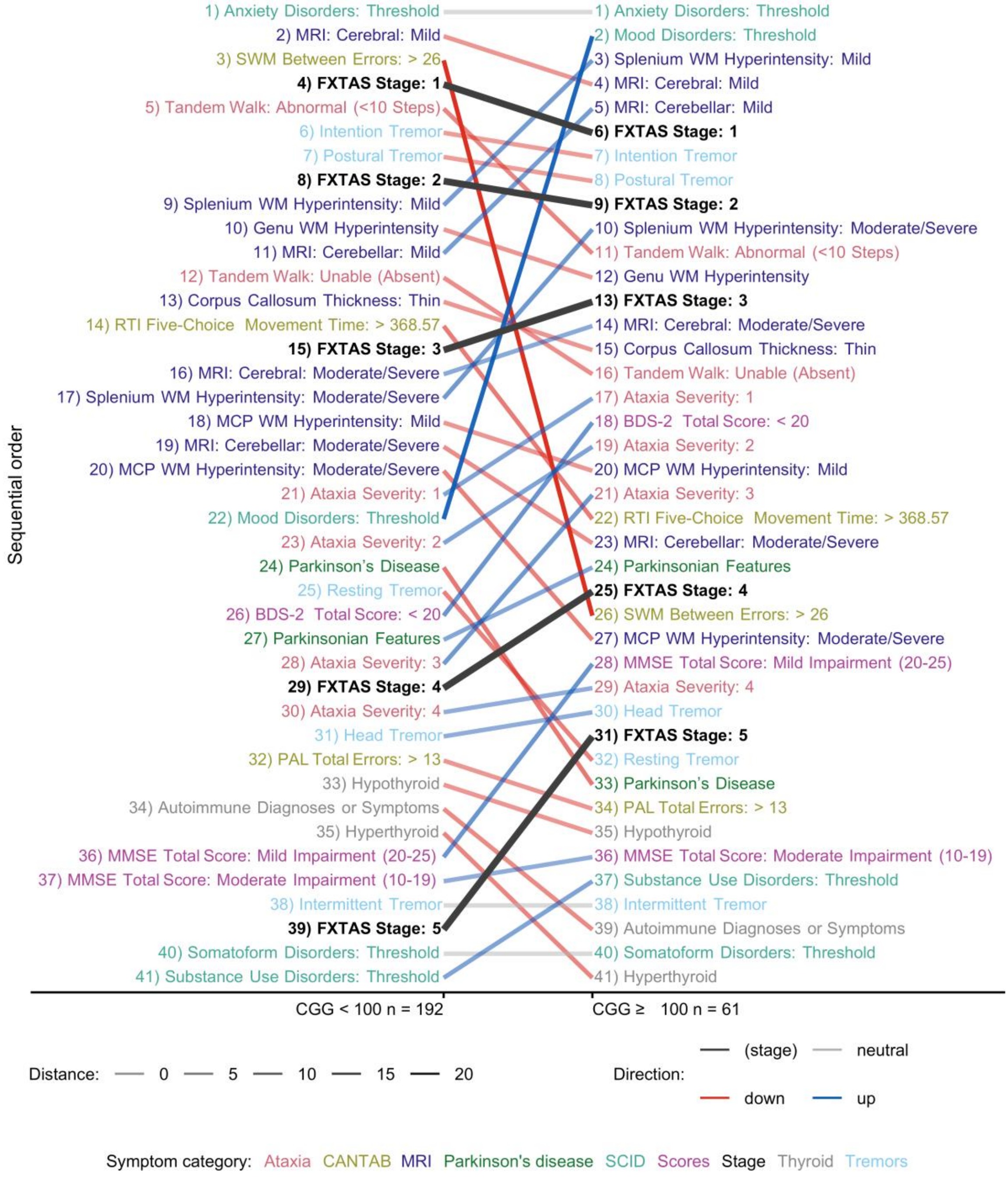

Comparing CGG levels stratified by sex

Supplementary Fig. 8: **Event sequences stratified by CGG repeats, among males.** The heatmap colors (violet red, royal blue, cadet blue, khaki, forest green) indicate the ordinal levels of symptom progression (Table 2). Heatmap color gradient intensity represents the Bayesian posterior probability of the sequence position; the brighter the color, the more probable that the corresponding symptom event occurs in that position in the sequence. Label text colors indicate symptom categories (Table 2). Abbreviations: BDS-2 = Behavior Dyscontrol Scale - Second Edition; CANTAB = Cambridge Neuropsychological Test Automated Battery; FXTAS = Fragile X-associated tremor/ataxia syndrome; MCP = Middle cerebellar peduncle; MMSE = Mini-Mental State Exam; Mod. = Moderate; MRI = Magnetic Resonance Imaging; PAL = Paired Associates Learning; RTI = Reaction Time; SCID = Structured Clinical Interview for DSM Disorders; SWM = Spatial Working Memory. Permutation test statistic (log-likelihood): -3139.7; p-value = 0.380 (N = 152 premutation carriers).

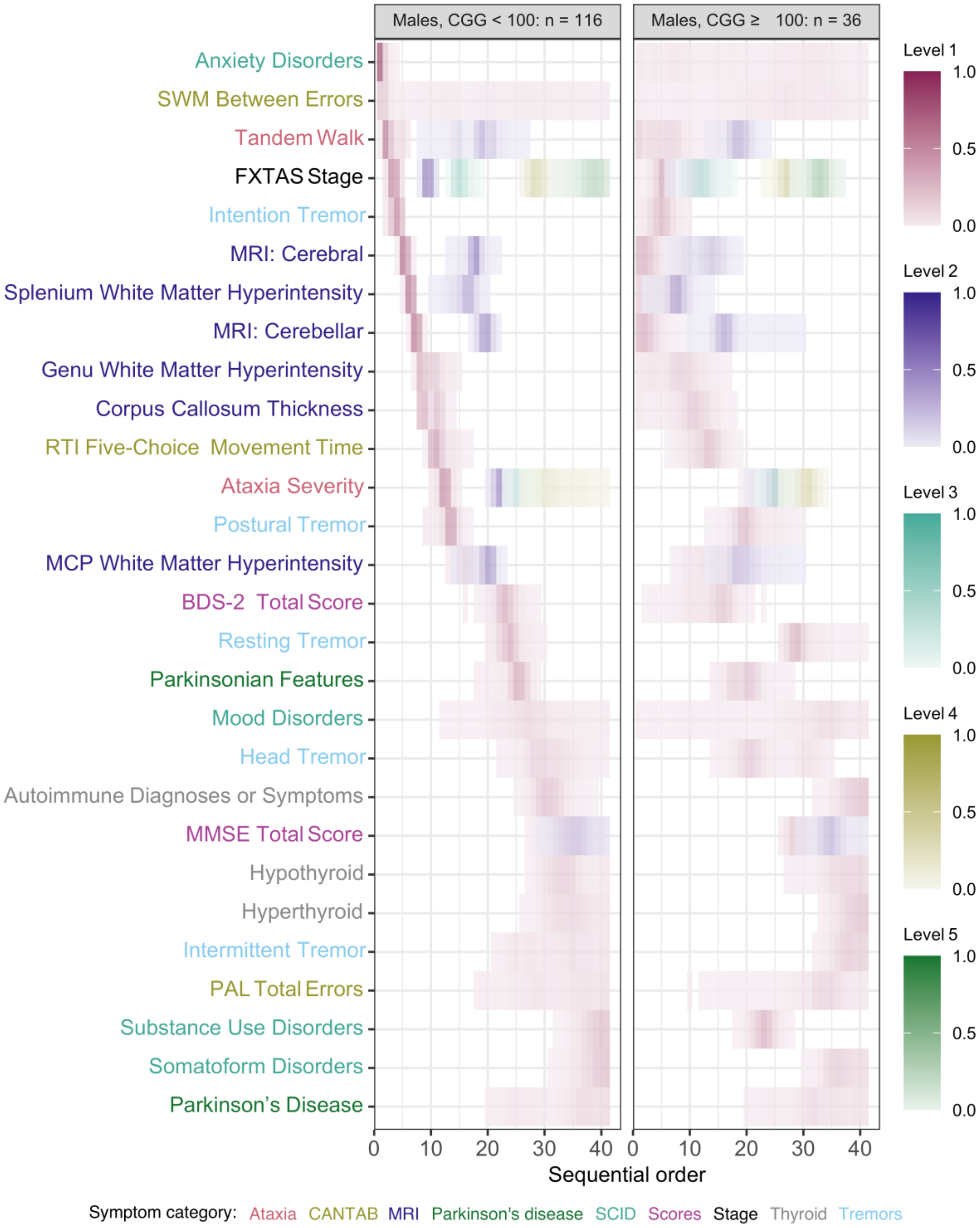

Supplementary Fig. 9: **Positional differences in estimated event sequence between CGG repeat levels, among males**. Red lines indicate symptoms that moved to later positions between the left-hand subgroup and the right-hand subgroup. Blue lines indicate symptoms that moved to earlier positions. Light gray lines indicate symptoms that did not change positions. Black lines indicate FXTAS stages. Line opacity levels indicate the number of positions changed (higher opacity represents more positions changed). Label text colors indicate symptom categories (Table 2). Yellow highlights indicate symptoms with clinically-significant positional differences between subgroups. Abbreviations: BDS-2 = Behavior Dyscontrol Scale - Second Edition; CANTAB = Cambridge Neuropsychological Test Automated Battery; FXTAS = Fragile X-associated tremor/ataxia syndrome; Hyp. = Hyperintensity; MCP = Middle cerebellar peduncle; MMSE = Mini-Mental State Exam; Mod. = Moderate; MRI = Magnetic Resonance Imaging; PAL = Paired Associates Learning; RTI = Reaction Time; SCID = Structured Clinical Interview for DSM Disorders; SWM = Spatial Working Memory; WM = White Matter. Permutation test statistic (log-likelihood): -3139.7; p-value = 0.380 (N = 152 premutation carriers).

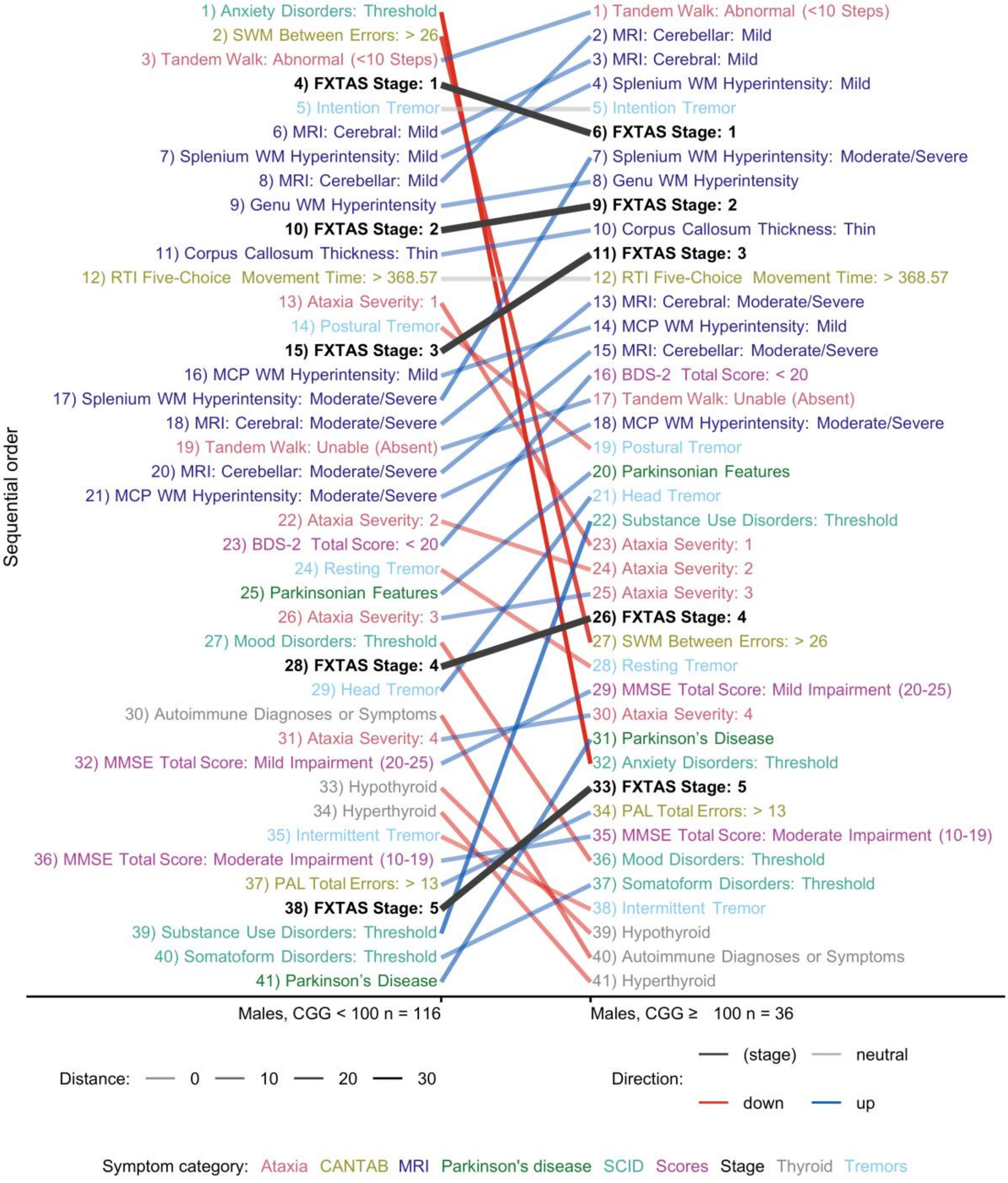

Supplementary Fig. 10: **Event sequences stratified by CGG repeats (<100 vs 100+), among females.** The heatmap colors (violet red, royal blue, cadet blue, khaki, forest green) indicate the ordinal levels of symptom progression (Table 2). Heatmap color gradient intensity represents the Bayesian posterior probability of the sequence position; the brighter the color, the more probable that the corresponding symptom event occurs in that position in the sequence. Label text colors indicate symptom categories (Table 2). Abbreviations: BDS-2 = Behavior Dyscontrol Scale - Second Edition; CANTAB = Cambridge Neuropsychological Test Automated Battery; FXTAS = Fragile X-associated tremor/ataxia syndrome; MCP = Middle cerebellar peduncle; MMSE = Mini-Mental State Exam; Mod. = Moderate; MRI = Magnetic Resonance Imaging; PAL = Paired Associates Learning; RTI = Reaction Time; SCID = Structured Clinical Interview for DSM Disorders; SWM = Spatial Working Memory. Permutation test statistic (log-likelihood): -2118.52; p-value = 0.730 (N = 101 premutation carriers).

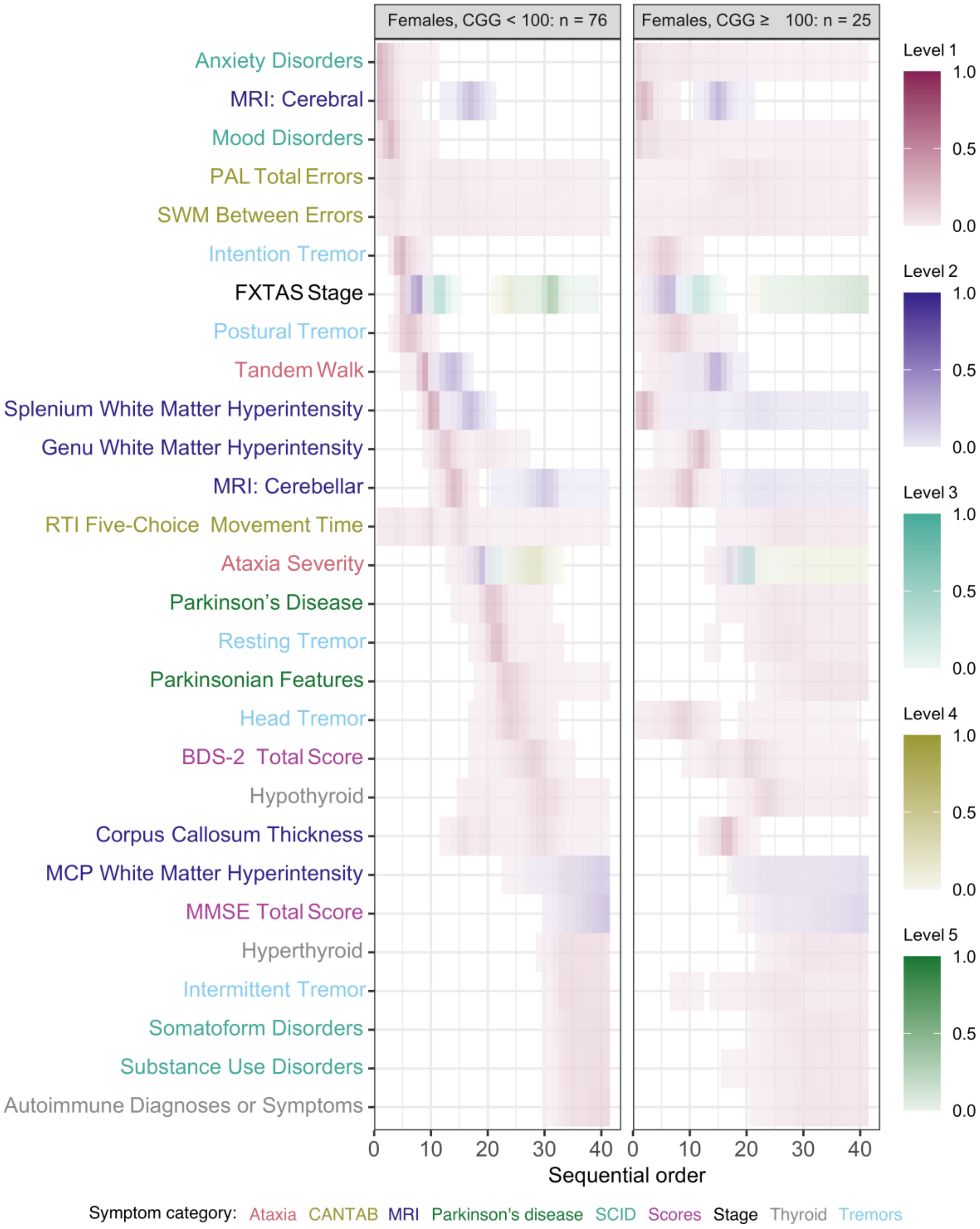

Supplementary Fig. 11: **Positional differences in estimated event sequence between CGG repeats (<100 vs 100+)**. Red lines indicate symptoms that moved to later positions between the left-hand subgroup and the right-hand subgroup. Blue lines indicate symptoms that moved to earlier positions. Light gray lines indicate symptoms that did not change positions. Black lines indicate FXTAS stages. Line opacity levels indicate the number of positions changed (higher opacity represents more positions changed). Label text colors indicate symptom categories (Table 2). Yellow highlights indicate symptoms with clinically-significant positional differences between subgroups. Abbreviations: BDS-2 = Behavior Dyscontrol Scale - Second Edition; CANTAB = Cambridge Neuropsychological Test Automated Battery; FXTAS = Fragile X-associated tremor/ataxia syndrome; Hyp. = Hyperintensity; MCP = Middle cerebellar peduncle; MMSE = Mini-Mental State Exam; Mod. = Moderate; MRI = Magnetic Resonance Imaging; PAL = Paired Associates Learning; RTI = Reaction Time; SCID = Structured Clinical Interview for DSM Disorders; SWM = Spatial Working Memory; WM = White Matter. Permutation test statistic (log-likelihood): -2118.52; p-value = 0.730 (N = 101 premutation carriers).

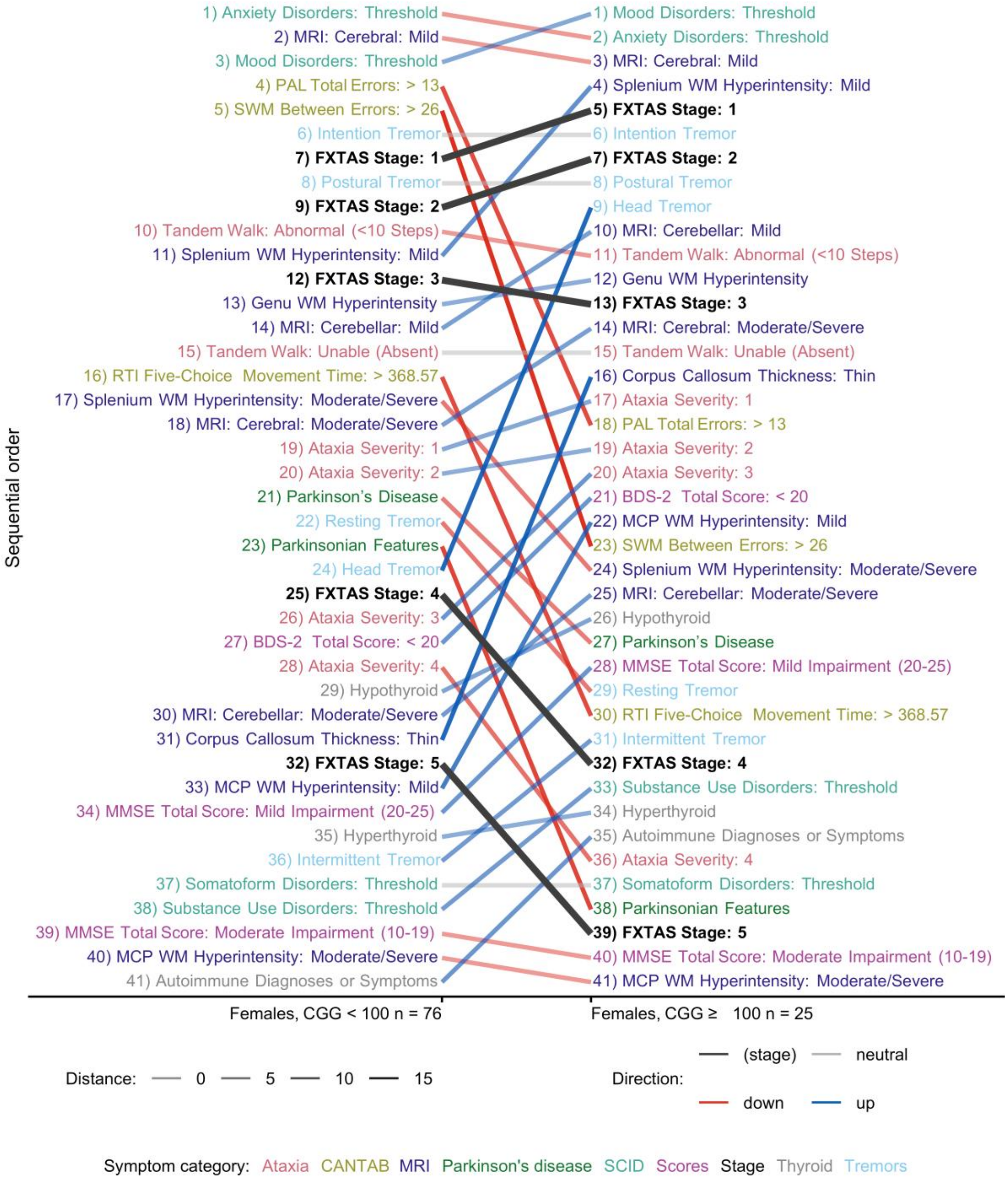

Detecting latent subtypes

The Ordinal SuStaIn algorithm clusters observations into subgroups based on their shared compatibility with particular event sequences. As a simplified example, if participants A, B, and C all had experienced ataxia but no tremors by the time when they were assessed for the study, and participants D, E, and F had all experienced tremors but not ataxia, then participants A, B, and C would be grouped as a subgroup cluster in which ataxia precedes tremor, and participants D, E, and F would be clustered into a "tremor before ataxia" subgroup. If participants G, H, and I displayed both ataxia and tremors at the time of assessment, then the Ordinal SuStaIn algorithm might be unable to confidently determine which latent subgroup they belonged to, at least on the basis of these two symptoms. However, their other symptoms help the algorithm refine the clustering. On the other hand, if participants J, K, and L displayed neither ataxia nor tremors, they would also not be classified into either of the two clusters described above and would be assigned to a new cluster "Subgroup 0", representing individuals whose disease has not yet begun to progress by the time when they were assessed for the study.

To cluster observations in a given data set into latent subtypes using the Ordinal SuStaIn modeling algorithm, we determined the optimal number of clusters based on the Cross-Validation Information Criterion (CVIC) described in Young et al..[6] We also evaluated the consistency of our cross-validation procedure by looking at the distribution of out-of-fold log-likelihood ("OOFLL") across cross-validation folds (Young et al.).[6] Supplementary Fig. 13 shows the distribution of the OOFLL statistic as a function of number of latent subgroups.

Supplementary Fig. 12: **Selection criteria for number of latent subtypes: cross-validation information criterion (CVIC)**. Each data point represents the estimated CVIC (y-axis) for the model fit with a given number of latent subtypes (x-axis) on the full data set (N = 253 carriers and 44 controls).

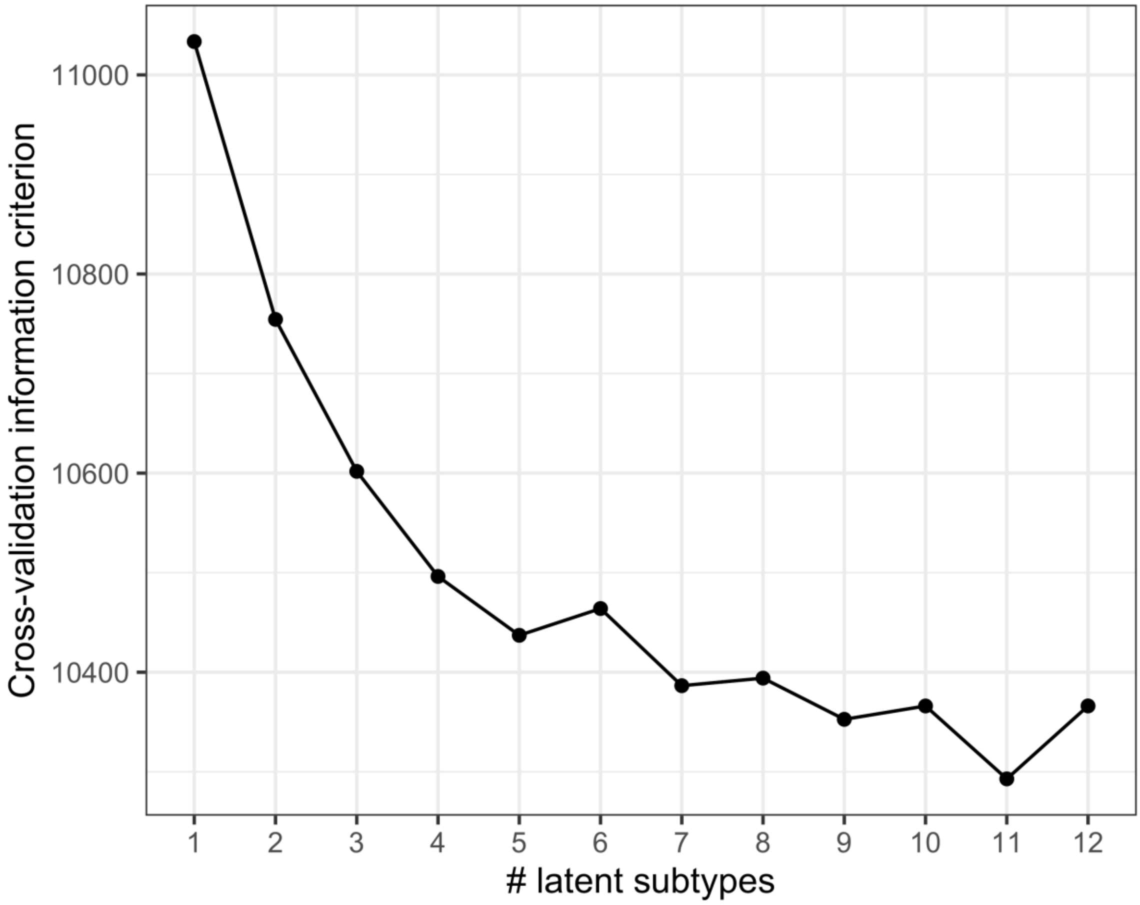

Supplementary Fig. 13: **Selection criteria for number of latent subtypes: Out-Of-Fold Log-Likelihood criterion (OOFLL)**. Each data point represents the OOFLL statistic (i.e., the test set log-likelihood across cross-validation (CV) folds) for the Ordinal SuStaIn model fit with a given number of latent subtypes on the full data set (N = 253 carriers and 44 controls).

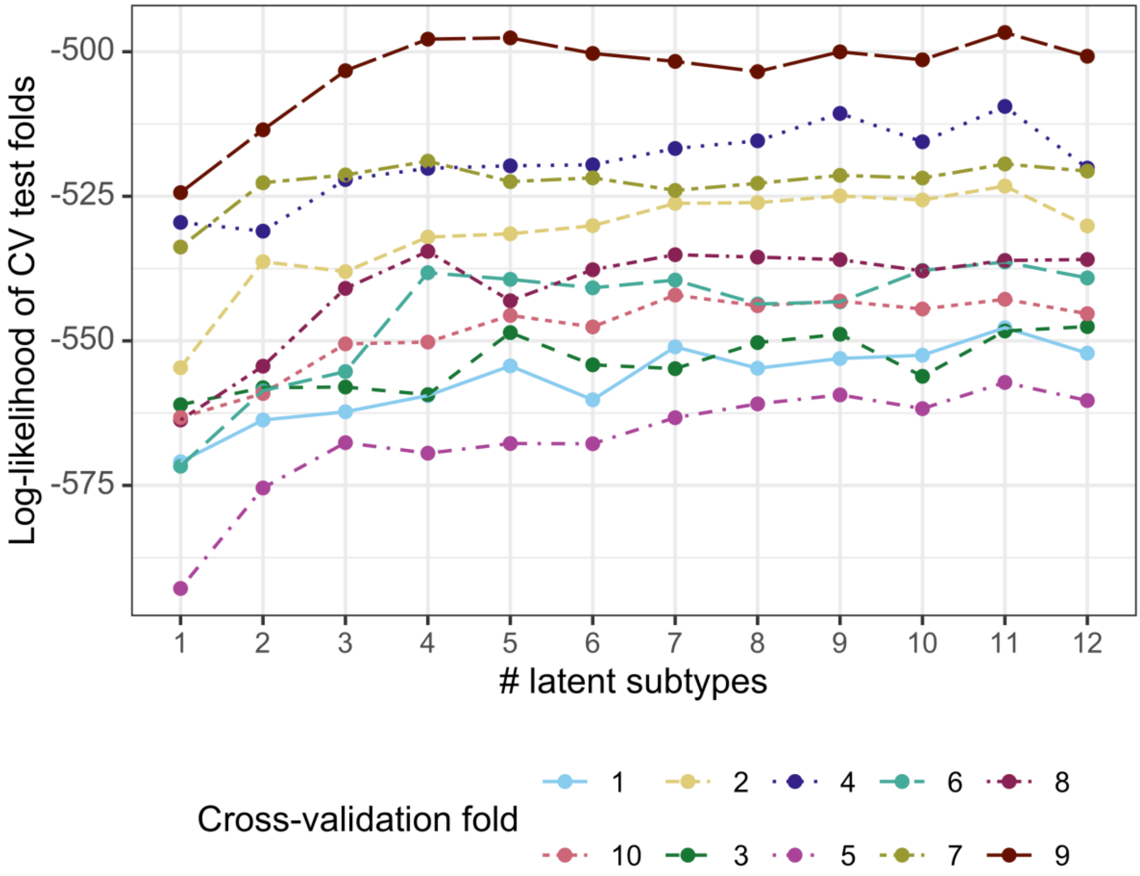

Supplementary Fig. 14: **Event sequences for four latent subtypes.** The heatmap colors (violet red, royal blue, cadet blue, khaki, forest green) indicate the ordinal levels of symptom progression (Table 2). Heatmap color gradient intensity represents the Bayesian posterior probability of the sequence position; the brighter the color, the more probable that the corresponding symptom event occurs in that position in the sequence. Label text colors indicate symptom categories (Table 2). Abbreviations: BDS-2 = Behavior Dyscontrol Scale - Second Edition; CANTAB = Cambridge Neuropsychological Test Automated Battery; FXTAS = Fragile X-associated tremor/ataxia syndrome; MCP = Middle cerebellar peduncle; MMSE = Mini-Mental State Exam; Mod. = Moderate; MRI = Magnetic Resonance Imaging; PAL = Paired Associates Learning; RTI = Reaction Time; SCID = Structured Clinical Interview for DSM Disorders; SWM = Spatial Working Memory. Mean CGG repeats differed significantly between subtypes; one-way ANOVA F test statistic: 3.13 (DFs: 3 and 101.2); p-value = 0.029 (N = 214 premutation carriers; 39 carriers had experienced too few events to be accurately classified into a subtype and were excluded from these results).

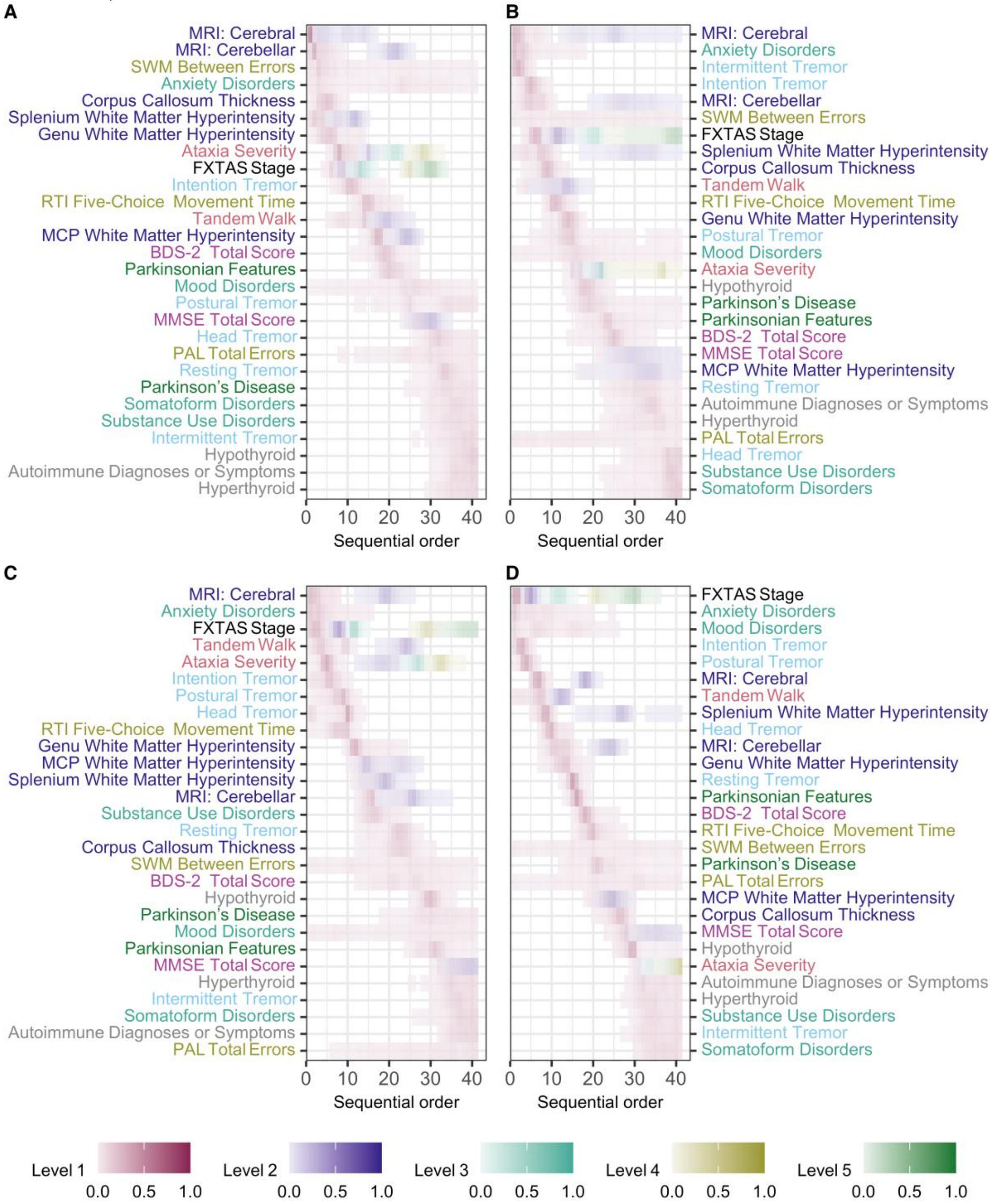

Supplementary Fig. 15: **Distribution of estimated disease stage, stratified by latent subtype**. Red horizontal line indicates minimum sample size recommended for the Ordinal SuStaIn algorithm, three observations per stage.[2] Bar heights represent the number of premutation carriers (y-axis) clustered into each combination of subtype (panel) and stage (x-axis).

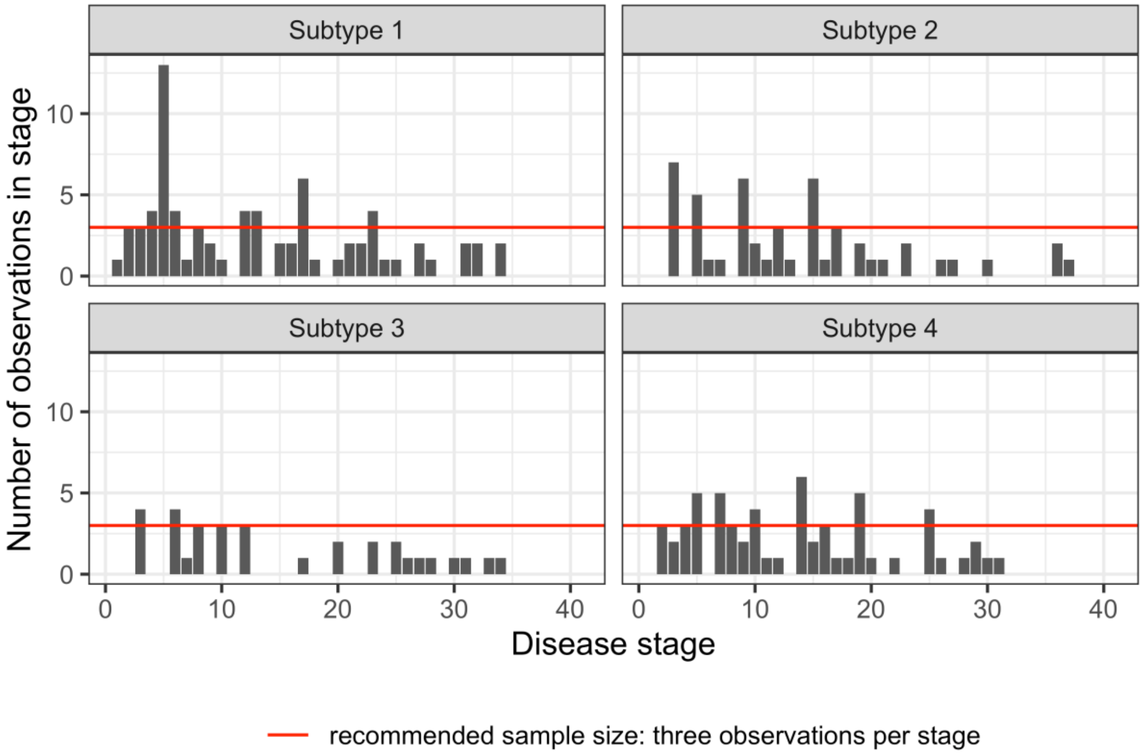

References

1. Talebi M, Majdi A, Kamari F, Sadigh-Eteghad S. The Cambridge Neuropsychological Test Automated Battery (CANTAB) versus the Minimal Assessment of Cognitive Function in Multiple Sclerosis (MACFIMS) for the assessment of cognitive function in patients with multiple sclerosis. *Multiple Sclerosis and Related Disorders*. 2020;43:102172. https://doi.org/10.1016/j.msard.2020.102172

2. Young AL, Vogel JW, Aksman LM, et al. Ordinal SuStaIn: Subtype and stage inference for clinical scores, visual ratings, and other ordinal data. *Frontiers in artificial intelligence*. 2021;4:613261. https://doi.org/10.3389/frai.2021.613261

3. Greco CM, Berman RF, Martin RM, et al. Neuropathology of fragile X-associated tremor/ataxia syndrome (FXTAS). *Brain*. 2006;129(1):243-255. doi:10.1093/brain/awh683

4. Leehey MA, Berry-Kravis E, Goetz CG, et al. FMR1 CGG repeat length predicts motor dysfunction in premutation carriers. *Neurology*. 2008;70(16_part_2):1397-1402. doi:10.1212/01.wnl.0000281692.98200.f5

5. Tassone F, Protic D, Allen EG, et al. Insight and recommendations for fragile X-premutation-associated conditions from the fifth international conference on FMR1 premutation. *Cells*. 2023;12(18):2330.

6. Young AL, Marinescu RV, Oxtoby NP, et al. Uncovering the heterogeneity and temporal complexity of neurodegenerative diseases with Subtype and Stage Inference. *Nature communications*. 2018;9(1):4273. doi:10.1038/s41467-018-05892-0
